# Association of multimorbidity and disease clusters with neuroimaging and cognitive outcomes in UK Biobank

**DOI:** 10.1016/j.tjpad.2025.100208

**Published:** 2025-05-26

**Authors:** Shehab Uddin Al Abid, Catherine M Calvin, Danial Qureshi, Michele Veldsman, Elżbieta Kuźma, Thomas J. Littlejohns

**Affiliations:** a Nuffield Department of Population Health, University of Oxford, Oxford, OX3 7LF, UK; b Health Data Research UK, University of Oxford (HDRUK-Oxford), Oxford, UK; c UK Biobank, Nuffield Department of Population Health, University of Oxford, Oxford, OX3 7LF, UK; d PhD2B-Limited, Oxford, UK; e Albertinen Krankenhaus/Albertinen Haus gGmbH, Academic Teaching Hospital of the Faculty of Medicine, University of Hamburg, Hamburg, Germany

**Keywords:** Multimorbidity, Disease clusters, Neuroimaging, Cognitive function, Brain health

## Abstract

**Background:**

The relationship between multimorbidity, particularly disease clusters, with neuroimaging and cognitive outcomes that typically manifest prior to clinical diagnosis of dementia, remains understudied. This study investigated whether multimorbidity is associated with dementia-related neuroimaging and cognitive outcomes in the UK Biobank cohort.

**Methods:**

This cross-sectional study used data from UK Biobank participants who attended imaging assessments between 2014–2023, and were free from neurological conditions, including dementia. Multimorbidity was defined as the coexistence of two or more long-term conditions, selected from a standardised criteria of 39 conditions. Latent class analyses were used to identify disease clusters. Neuroimaging outcomes were measured using magnetic resonance imaging, and cognition was assessed by seven tests measuring different cognitive domains. Multivariable linear regression was used to assess the association between multimorbidity and disease clusters with neuroimaging and cognitive outcomes.

**Results:**

A total of 43,160 participants were included (mean [standard deviation] age, 64.2 [7.7] years, 53.1 % female). Multimorbidity was present among 14,339 (33.2 %) participants, and was associated with reduced grey matter volume, total brain volume, left hippocampal volume, increased cerebrovascular pathology as well as reduced domain-specific cognitive function. A strong dose-response relationship was observed with the increasing number of multimorbid conditions across these outcomes. A disease cluster driven by cardiometabolic conditions was consistently associated with poorer brain health across all outcomes. Disease clusters driven by respiratory, mental health and other conditions showed less consistent associations.

**Conclusions:**

Multimorbidity was strongly associated with poorer brain health, particularly within the cardiometabolic disease cluster. Given that UK Biobank participants are, on average, healthier than the general population, future studies in more diverse and representative cohorts would be valuable.

## 1 Introduction

Worldwide, one in three adults live with two or more long-term health conditions, a state referred to as multimorbidity [1]. The prevalence of multimorbidity increases substantially with age, affecting more than 50 % of global population aged 60 and above [2]. Given the rapidly ageing global population, multimorbidity has emerged as a critical public health challenge, with some experts identifying it as the next global pandemic [3]. While individual conditions such as hypertension, diabetes, and depression are recognized risk factors for dementia, there is growing evidence that multimorbidity may compound this risk even further [[4], [5], [6], [7]]. However, dementia has a long pre-clinical phase, during which subtle neurodegenerative and cognitive changes may occur years before a clinical diagnosis [8,9]. Understanding how multimorbidity relates to neurodegeneration, cerebrovascular pathology, and cognitive decline is therefore crucial to elucidating the pathways that link multimorbidity with dementia risk.

Multimorbidity has previously been associated with poorer brain health based on either neuroimaging measures, such as lower hippocampal volume, or reduced global cognitive function [[10], [11], [12]]. However, to our knowledge, no study has comprehensively examined both neuroimaging and cognitive outcomes within the same population, nor has any prior work employed a wide range of biomarkers to capture the multifaceted nature of brain health. Furthermore, much of the existing evidence is derived from small-scale studies, which are susceptible to random variation and may not provide robust estimates of association [10,11,13]. Moreover, these studies have often conceptualised multimorbidity as a binary variable, overlooking the fact that the specific combinations or clusters of conditions may differentially impact brain health [14].

To address these knowledge gaps, we investigated the association between multimorbidity and a range of neuroimaging and cognitive outcomes of brain health in a large, population-based sample of middle- to older-aged adults without dementia or other neurological conditions. We also examined whether disease clusters influence these associations, providing a more nuanced understanding of how specific multimorbidity patterns affect brain health.

## 2 Method

### 2.1 Study population

UK Biobank (UKB) is a large population-based study of approximately 0.5 million participants aged 40–69 years recruited from England, Scotland, and Wales between 2006 and 2010 [15]. Beginning in 2014, UKB set up an ongoing multi-modal imaging assessment with assessment centres located in Newcastle, Stockport, Reading and Bristol [16]. Participants who were deemed unsafe for undergoing magnetic resonance imaging (MRI) due to factors such as presence of metals, electrical implants or recent surgery were excluded.

During the imaging assessment, participants provided socio-demographic, lifestyle, and health information through a touchscreen questionnaire and nurse-led verbal interview, underwent physical examinations, and provided blood samples which were used to perform genome-wide genotyping [17]. In the current cross-sectional study, we included 45,001 participants who attended the imaging assessment. We excluded participants with self-reported pre-existing neurological conditions (*n* = 1008), outlier variables (*n* = 238), and missing covariate information (*n* = 595), resulting in a final sample of 43,160 participants (**Supplemental Fig. 1 and Supplemental Table 1**).

UKB received ethical approval from the National Health Service North West Centre for Research Ethics Committee (Ref: 11/NW/0382). All participants provided electronically signed informed consent.

### 2.2 Multimorbidity

Participants self-reported their medical conditions during a nurse-led verbal interview at the imaging assessment visit. The interviewer used a hierarchical framework broadly based on the International Statistical Classification of Diseases and Related Health Problems, Tenth Revision (ICD-10), to ensure standardised data entry [18]. In this study, the selection of diseases for defining multimorbidity was based on the list developed by Barnett and colleagues (**Supplemental Table 2**) [19]. Multimorbidity was defined as the presence of ≥2 long-term health conditions from a list of 39 health conditions [4]. Participants with none or only one condition were categorized as not having multimorbidity and formed the reference group in all analyses.

### 2.3 Neuroimaging

Neuroimaging was conducted using Siemens Skyra 3T MRI scanners (software VD13) with a standard 32-channel head coil. High-resolution T1-weighted and three-dimensional T2-weighted fluid-attenuated inversion recovery (FLAIR) images were acquired. Imaging-derived phenotypes were obtained from the raw T1- and T2-weighted brain MRI scans using an automated image-processing pipeline developed by an external group in collaboration with the UK Biobank. This pipeline applied standardised pre-processing with FSL (version 5.0.10) and FreeSurfer (version 6.0), along with several quality control measures [20,21].

In this study, we selected seven neuroimaging outcomes based on their relevance to cognitive decline and dementia [22,23]. These measures included global brain volumes (grey matter, white matter, total brain); hippocampal volumes (left hippocampal, right hippocampal, total hippocampal); and white matter hyperintensity volume (**Supplemental Table 3**). Total brain volume was derived by summing grey and white matter volume, and total hippocampal volume was derived by summing right and left hippocampal volume. White matter volume represents the total volume of white matter in the brain, whereas white matter hyperintensities (WMH) refer to regions of white matter that appear brighter on brain MRI scans. WMH are commonly associated with ageing, cognitive decline, and dementia [24,25]. WMH volume was natural log-transformed due to a positively skewed distribution. We used the median absolute deviation method to identify and exclude outliers (*n* = 222, <0.6 % of the whole sample) [24,26]. Lower global and hippocampal brain volumes are indicative of poorer brain health, whereas lower white matter hyperintensity volume indicates better brain health [25,27].

### 2.4 Cognitive function

We selected seven cognitive tests administered at the UK Biobank imaging assessment, evaluating distinct cognitive domains. These measures included: executive function (trail-making tests [TMT]. A and B); verbal and numerical reasoning (fluid intelligence test); verbal declarative memory (paired associate learning task); non-verbal reasoning (matrix pattern completion task); numerical memory (backward digit span task); and processing speed (symbol-digit substitution task). A higher score on the TMTs indicates poorer cognition (as they measure the time required to complete the tasks), whereas higher scores indicate better cognition for the remaining tests [28]. TMT scores were natural log-transformed due to a positively skewed distribution. Detailed descriptions of the cognitive tests used in the UK Biobank have been published elsewhere [28]. Complete information on the cognition variables used in this study is provided in the **Supplemental Methods** and in **Supplemental Table 3**.

### 2.5 Covariates

Covariates in the main model included age, sex (‘female’, ‘male’), education (‘primary’, ‘secondary’, ‘post-secondary non-tertiary’, ‘tertiary’); socioeconomic status (quintiles) measured using the Townsend deprivation index, which combines information on social class, employment, car availability, and housing [29]; and, self-reported ethnicity (‘white’, ‘non-white’). A single 'non-white' ethnic group was used because only a small proportion of participants (<5 %) identified as Asian or Asian British, Black or Black British, Chinese, mixed ethnicity, or other ethnic group. Additionally, lifestyle factors such as smoking status (‘never’, ‘former’, ‘current’), alcohol intake (‘never’, ‘former’, ‘current’), and body mass index (BMI; kg/m^2^) were further accounted for in secondary analyses [4]. Full details of all covariates used are provided in **Supplemental Table 4**.

### 2.6 Genetics

Apolipoprotein E (APOE) ε4 carrier status was derived using the rs429358 and rs7412 single nucleotide polymorphisms (SNPs) [30]. Dementia polygenic risk score (PRS) was based on a 39-SNP score (available on the Polygenic Score Catalog as PGS001775) developed on external genome-wide association studies data [31]. PLINK 2 with a hard call threshold of 0.1 was used to ensure that none of the SNPs were in linkage disequilibrium with the APOE SNPs (R2 < 0.3). One SNP had a minor allele frequency of <0.005 and was excluded, resulting in a final score of 38 SNPs. All SNPs had imputation information >0.9, and none were ambiguous [31]. The dementia PRS was initially divided into quintiles and subsequently grouped into three categories: 'low' (quintile 1), 'intermediate' (quintiles 2–4), and 'high' (quintile 5), with higher scores indicating an increased risk of dementia [32].

### 2.7 Statistical analysis

Descriptive statistics were used to compare characteristics between participants with and without multimorbidity. Separate multivariable linear regression models were used to examine the association of multimorbidity with neuroimaging and cognitive outcomes. Analyses were adjusted for age, sex, age-squared, age*sex, education, socio-economic status, ethnicity, and assessment centre [33,34]. In line with previous recommendations from the UK Biobank neuroimaging studies, we included quadratic age terms (age²) in our models to account for non-linear age effects across all outcomes [33,35]. Furthermore, age-sex interaction terms were included in all models to account for their joint effects on brain health, as age and sex interact non-additively in the context of brain health [33,35]. Head size and scanner position were additionally adjusted for in the neuroimaging analyses [33]. Lifestyle factors such as smoking status, alcohol intake, and BMI were not included in the main model due to their potential role in the causal pathway between multimorbidity and brain health [4]. However, to determine the impact of adjusting for lifestyle factors, a secondary analysis was conducted by repeating the main analyses with additional adjustments for these variables. All outcomes were transformed into standardised scores, with a mean of 0 and a standard deviation of 1, to facilitate comparison of effect size across results. Dose-response associations were examined using separate models, categorising multimorbidity into groups of 0–1, 2, 3, and ≥4 conditions.

Previous studies on multimorbidity and dementia have shown differences in the strength of associations based on age, sex, and genetic factors [4,6,32]. Therefore, using a hypothesis-free approach we also explored whether the associations of multimorbidity with neuroimaging and cognitive outcomes were modified by these factors. Genetics is a major risk factor for dementia, with evidence indicating they interact with other risk factors, thereby modifying their effects [4]. To address this, we conducted a comprehensive examination of genetic interactions in the context of multimorbidity and brain health, incorporating both APOE ε4 status and non-APOE polygenic risk scores (PRS) for dementia [31]. To investigate effect modification, interaction terms with multimorbidity were entered into models for the following factors: (1) APOE ε4 carrier (non- ε4 carrier, ε4 carrier); dementia PRS (low, intermediate, high), (2) age (≤65, >65 years), and (3) sex (female, male). PRS interactions were restricted to individuals who reported their ethnicity as ‘white’, as the dementia PRS is specific to this population [4,32].

Latent class analysis (LCA) was used to identify disease clusters. Participants with multimorbidity were assigned to a single, non-overlapping cluster, while health conditions could contribute to multiple clusters with varying probabilities [4]. Separate cluster estimations for males and females were not conducted, as exploratory analyses of the total sample did not identify any clusters predominantly associated with one sex [4]. A random training sample of 70 % of participants with multimorbidity (*n* = 10,038) was used to determine the optimal number of clusters and to estimate the association of disease clusters with neuroimaging and cognitive outcomes. Output statistics were generated for multiple LCA models, ranging from 1 to 12 cluster solutions. The optimal number of clusters was determined using a combination of sample size-adjusted Bayesian Information Criteria (ABIC) statistics, Consistent Akaike Information Criterion (CAIC), entropy, clinical judgment, and capping the smallest cluster to greater than 5 % of the training sample [4,36]. Lower ABIC and CAIC values indicate better-fitting models, whereas higher entropy values indicate improved classification accuracy. Each cluster was characterised by the three health conditions with the highest probabilities, each exceeding 5 %, of contributing to that specific cluster. The observed prevalence of conditions that characterised the clusters needed to be more than that of the expected prevalence in the training sample [4]. To assess the validity of the clusters, the remaining 30 % of participants (*n* = 4,301) with multimorbidity (test sample) had their conditions entered into LCA models, with the number of clusters set to match the optimal number identified in the training set. The characterisation and relative size of the clusters determined from the training and test samples were compared, as were their associations with all outcomes in the linear regression models.

All models adhered to linear regression assumptions. Participants with missing data or those answering ‘do not know’ or 'prefer not to answer' constituted <1.5 % of the sample, and were excluded. Given the hypothesis-driven nature of our analysis, we did not adjust for multiple comparisons to minimize the risk of Type-II error, based on prior evidence linking multimorbidity and disease clusters to an increased risk of dementia [4,[37], [38], [39]]. All P-values were two-sided, with statistical significance set at <0.05. Analyses were performed using Stata version 17 (Stata Corp, TX, United States) and R version 4.3.0 (R Core Team, Vienna, Austria).

## 3 Results

A total of 43,160 participants were included, of whom 14,339 (33.2 %) had multimorbidity. Among the 28,821 participants without multimorbidity, 16,344 (56.7 %) had no long-term conditions, while 12,477 (43.3 %) had one long-term condition. Overall, mean age was 64.2 (standard deviation [SD]=7.7) years, with 53.1 % being female. Participants with multimorbidity were more likely to be older, of White ethnicity, and have lower educational qualifications. There were no differences in APOE-ε4 carrier status or dementia polygenic risk score (PRS) between those with and without multimorbidity (Table 1). Amongst all participants, hypertension was the most common condition (20.2 %), followed by painful conditions (18.5 %), and cancer (10.8 %) (**Supplemental Table 2**)**.**

**Table 1:** Study characteristics, overall and by presence of multimorbidity.

Characteristics	No Multimorbidity	Multimorbidity	Total	*P*-value
	*N* = 28,821	*N* = 14,339	*N* = 43,160	
**Age, years**	63.5 (7.7)	65.7 (7.5)	64.2 (7.7)	<0.001
**Sex**				0.80
Female	15,322 (53.2 %)	7604 (53.0 %)	22,926 (53.1 %)	
Male	13,499 (46.8 %)	6735 (47.0 %)	20,234 (46.9 %)	
**Ethnic background**				<0.001
White	27,884 (96.7 %)	13,978 (97.5 %)	41,862 (97.0 %)	
Non-White	937 (3.3 %)	361 (2.5 %)	1298 (3.0 %)	
**Townsend deprivation index, quintiles**				0.22
1 (least deprived)	6884 (23.9 %)	3377 (23.6 %)	10,261 (23.8 %)	
2	6541 (22.7 %)	3372 (23.5 %)	9913 (23.0 %)	
3	5969 (20.7 %)	3015 (21.0 %)	8984 (20.8 %)	
4	5417 (18.8 %)	2643 (18.4 %)	8060 (18.7 %)	
5 (most deprived)	4010 (13.9 %)	1932 (13.5 %)	5942 (13.8 %)	
**Education**				<0.001
Primary	1631 (5.7 %)	1143 (8.0 %)	2774 (6.4 %)	
Secondary	3684 (12.8 %)	1977 (13.8 %)	5661 (13.1 %)	
Post-secondary non-tertiary	5178 (18.0 %)	2517 (17.6 %)	7695 (17.8 %)	
Tertiary	18,328 (63.6 %)	8702 (60.7 %)	27,030 (62.6 %)	
**Smoking status**				<0.001
Never	18,545 (64.3 %)	8479 (59.1 %)	27,024 (62.6 %)	
Former	9217 (32.0 %)	5345 (37.3 %)	14,562 (33.7 %)	
Current	982 (3.4 %)	446 (3.1 %)	1428 (3.3 %)	
**Alcohol intake**				<0.001
Never	883 (3.1 %)	504 (3.5 %)	1387 (3.2 %)	
Former	793 (2.8 %)	621 (4.3 %)	1414 (3.3 %)	
Current	27,135 (94.2 %)	13,210 (92.1 %)	40,345 (93.5 %)	
**Body Mass Index, Kg/m^2^**	26.1 (4.1)	27.4 (4.7)	26.5 (4.4)	<0.001
**APOE ε4 carrier status**^a^				0.069
Non-carrier	20,204 (73.8 %)	10,140 (74.6 %)	30,344 (74.1 %)	
ε4 carrier	7186 (26.2 %)	3445 (25.4 %)	10,631 (25.9 %)	
**Dementia PRS**^a^				0.008
Low	4618 (19.7 %)	2214 (19.3 %)	6832 (19.6 %)	
Intermediate	14,013 (59.9 %)	6922 (60.4 %)	20,935 (60.0 %)	
High	4784 (20.4 %)	2325 (20.3 %)	7109 (20.4 %)	
**Grey matter volume, ml**	616.2 (55.8)	606.5 (55.1)	612.9 (55.8)	<0.001
**White matter volume, ml**	545.1 (61.6)	542.3 (61.5)	544.1 (61.5)	<0.001
**Total brain volume, ml**	1161.3 (111.7)	1148.7 (110.0)	1157.1 (111.3)	<0.001
**WMH volume, ml**	2.7 (1.5, 5.4)	3.4 (1.8, 6.9)	2.9 (1.5, 5.9)	<0.001
**Left hippocampal volume, μl**	3782.7 (469.7)	3725.9 (470.7)	3763.8 (470.8)	<0.001
**Right hippocampal volume, μl**	3895.3 (481.3)	3846.3 (479.4)	3879.0 (481.2)	<0.001
**Total hippocampal volume, μl**	7678.1 (862.5)	7572.1 (862.7)	7642.9 (864.0)	<0.001
**Cognitive test scores**^a^				
Trail Making Test A	208 (177, 249)	215 (183,262)	210 (179, 253)	<0.001
Trail Making Test B	508 (410, 644)	536 (432,688)	517 (417, 658)	<0.001
Fluid Intelligence	6.6 (2.1)	6.5 (2.0)	6.6 (2.1)	<0.001
Paired Associate Learning	7.0 (2.6)	6.7 (2.7)	6.9 (2.6)	<0.001
Matrix Pattern Completion	8.0 (2.1)	7.8 (2.2)	8.0 (2.1)	<0.001
Backward Digit Span	6.8 (1.3)	6.7 (1.3)	6.8 (1.3)	<0.001
Symbol-digit Substitution	19.2 (5.2)	18.1 (5.3)	18.8 (5.3)	<0.001

*Notes:* Data are presented as mean (SD) for continuous measures (normally distributed), median (IQR) for non-normally distributed continuous variables and n (%) for categorical measures. *P-*values were derived from Pearson’s chi-squared test for categorical variables and linear regression for continuous variables.

For brain measures, lower white matter hyperintensity volume indicates better brain health, whereas lower volumes are indicative of poorer brain health for all other brain MRI measures. As for cognitive test scores, higher scores on the trail making test A or B indicate poorer cognition, while higher scores on the remaining tests indicate better cognition.

APOE= apolipoprotein E. CI= confidence interval. IQR= interquartile range. *N*= number of participants. PRS= polygenic risk score. SD= standard deviation. WMH= white matter hyperintensity.

a Genetic information and cognitive test scores were not available for all participants.

**Fig. 1:**
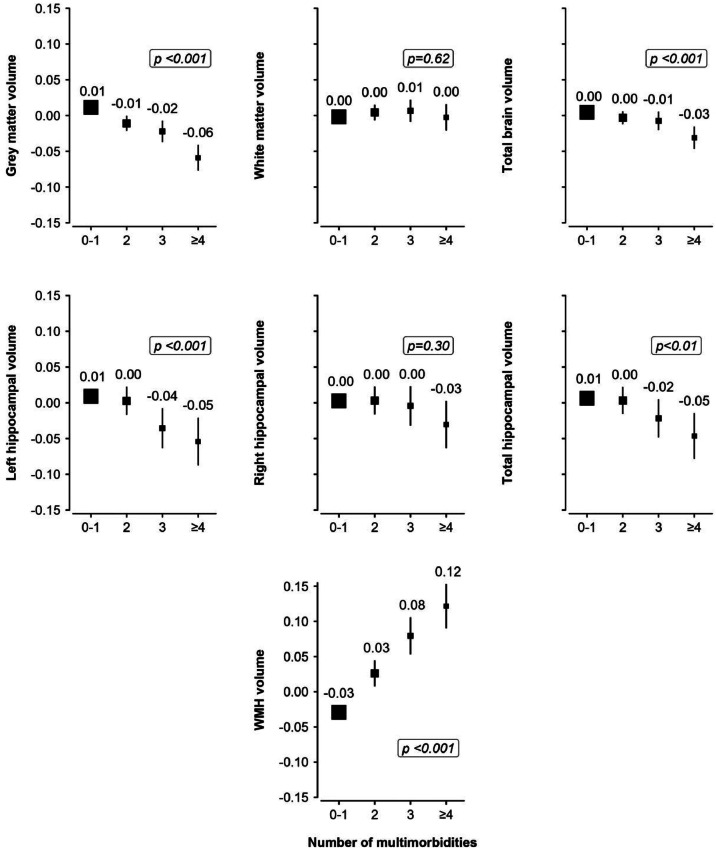
Multivariable linear regression analyses examining the associations between the number of multimorbidities and standardised neuroimaging outcomes.

**Fig. 2:**
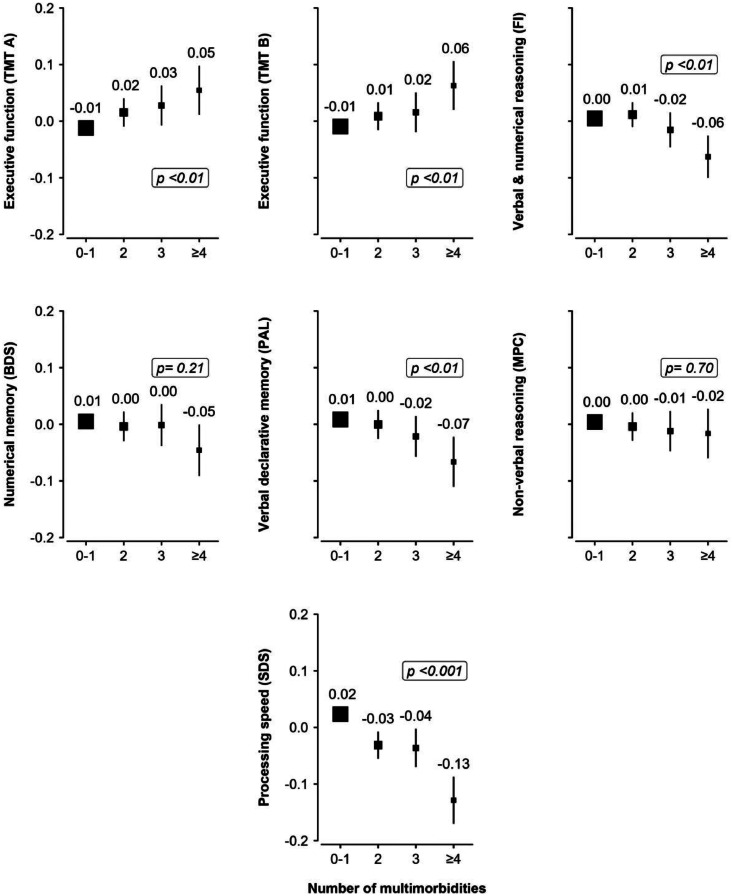
Multivariable linear regression analyses examining the associations between the number of multimorbidities and standardised cognitive outcomes.

Multimorbidity was associated with lower volumes of grey matter (β= −0.03 standard deviation (SD); 95 % confidence interval (CI): −0.04, −0.02), total brain (β= −0.01, 95 % CI: −0.02, −0.01), left hippocampus (β= −0.03; 95 % CI: −0.05, −0.01), total hippocampus (β= −0.02; 95 % CI: −0.04, 0.00), higher WMH volume (β= 0.09; 95 % CI: 0.07, 0.10), poorer executive function (β =0.04; 95 % CI: 0.02, 0.06), verbal declarative memory (β= −0.03; 95 % CI: −0.05, −0.01), and processing speed (β=−0.07; 95 % CI: −0.10, −0.05). Dose-response associations were observed between the number of multimorbid conditions and the aforementioned outcomes (Fig. 1, Fig. 2). There was no association between multimorbidity and white matter volume, right hippocampal volume, verbal and numerical reasoning, non-verbal reasoning and numerical memory (**Supplemental Fig. 2**). However, dose-response associations were observed between the number of multimorbid conditions and verbal and numerical reasoning (**Supplemental Table 6).** The associations with neuroimaging and cognitive outcomes remained similar when further adjusting for lifestyle factors, albeit the effect estimates were attenuated (**Supplemental Tables 5 and 7**)**.**

Lower WMH volume indicates better brain health, whereas lower volumes are indicative of poorer brain health for all other neuroimaging outcomes. WMH was log-transformed. All neuroimaging outcomes were standardised (mean = 0, standard deviation = 1), to facilitate comparison of effect sizes across outcomes. Standardised mean volumes of all brain measures are reported according to the number of multimorbidities. The regression models were adjusted for age, sex, age-squared, age*sex, assessment centre, scanner position, head size, education, Townsend deprivation index and ethnicity. Point estimates are plotted as squares, with the size of each square proportional to the inverse of the variance of the estimate. Horizontal lines represent 95 % CIs. For a better comparison, all Y-axes have been set to the same axis limit (−0.15 SD to 0.15 SD). P-shown indicates the p-value for the trend test across multimorbidity groups. CI=confidence interval. WMH = White matter hyperintensity.

Higher scores on the trail making test A or B indicate poorer cognition, while higher scores on the remaining tests indicate better cognition. Trail making tests A and B were log-transformed. All cognitive outcomes were standardised (mean = 0, standard deviation = 1), to facilitate comparison of effect sizes across outcomes. Standardised mean scores of all cognitive tests are reported according to the number of multimorbidities. The regression models were adjusted for age, sex, age-squared, age*sex, assessment centre, education, Townsend deprivation index and ethnicity. Point estimates are plotted as squares, with the size of each square proportional to the inverse of the variance of the estimate. Horizontal lines represent 95 % CIs. For a better comparison, all Y-axes have been set to the same axis limit (−0.20 SD to 0.20 SD). P-shown indicates the p-value for the trend test across multimorbidity groups. BDS= backward digit span task. CI= confidence interval. FI= fluid intelligence score. MPC= matrix pattern completion task. PAL= paired associate learning task. SD= standard deviation. SDS= symbol digit substitution task. TMT= trail making test.

Statistically significant interactions *(p* *<* *0.05*) were found between multimorbidity and dementia PRS for grey matter, white matter, and total brain volumes, with stronger associations observed among those with low or high PRS. No interactions were observed between multimorbidity status and APOE-ε4 carrier status (**Supplemental Fig. 3**). Multimorbidity significantly interacted with sex in association with grey matter volume and total brain volume (*p* < 0.001), with associations found only in males for grey matter (β=−0.07; 95 % CI: −0.08, −0.06) and total brain volume (β=−0.04; 95 % CI: −0.05, −0.02). No interactions between multimorbidity and age for any neuroimaging outcome were observed **(Supplemental Fig. 4)**.

For the cognitive outcomes, the only significant interaction was observed for TMT B by APOE-ε4 carrier status, and by sex (*p* < 0.05), with significant associations found only in APOE-ε4 carriers (β=0.08; 95 % CI: 0.04, 0.13) and in female (β=0.05; 95 % CI: 0.02, 0.08). No other significant interactions with genetics, sex or age were observed for other cognitive outcomes **(Supplemental Figs. 5 and 6)**.

Model selection statistics for the LCA are presented in **Supplemental Table 8** and **Supplemental Fig. 7**. When comparing LCA models with 2–12 classes, the 5-class solution was identified as the optimal number. This choice was based on the fact that it had optimum values for CAIC, ABIC and entropy and also represents a good balance between clinical interpretation and statistical concerns. There were no sex-dominant cluster groups, and certain clusters, though comprising a range of conditions, were largely defined by a single disease, such as asthma (**Supplemental Tables 9 and 10**). High similarity existed in the characteristics of the cluster groups between the training samples and the cluster groups in the test samples (**Supplemental Tables 11 and 12**).

**Fig. 3:**
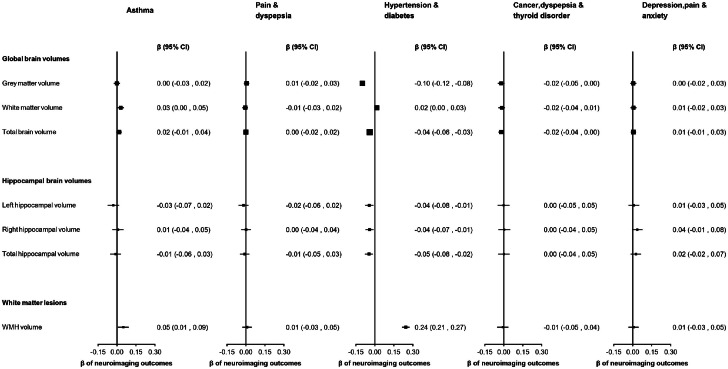
Multivariable linear regression analyses examining associations between disease clusters and standardised neuroimaging outcomes.

The 'hypertension and diabetes' cluster was the only cluster that was consistently associated with poorer neuroimaging outcomes (Fig. 3). The 'asthma' cluster was associated with higher WMH volume (β=0.05; 95 % CI: 0.01 to 0.09). The 'cancer, dyspepsia, and thyroid' cluster was associated with lower grey matter volume (β=−0.02; 95 % CI: −0.05, 0.00). The other clusters were not associated with any neuroimaging outcomes.

Lower WMH volume indicates better brain health, whereas lower volumes are indicative of poorer brain health for all other neuroimaging outcomes. WMH was log-transformed. All neuroimaging outcomes were standardised (mean = 0, standard deviation = 1), to facilitate comparison of effect sizes across outcomes. All regression models were adjusted for age, sex, age-squared, age*sex, assessment centre, scanner position, head size, education, Townsend deprivation index and ethnicity. Point estimates are plotted as squares, with the size of each square proportional to the inverse of the variance of the estimate. Horizontal lines represent 95 % CIs. Each disease cluster was characterised by the top 3 health conditions with the highest probabilities greater than 5 % of contributing to that cluster, excluding conditions for which observed prevalence was equal to or less than that of the total population’s expected prevalence. β= regression co-efficient. CI= confidence interval. WMH = White matter hyperintensity.

**Fig. 4:**
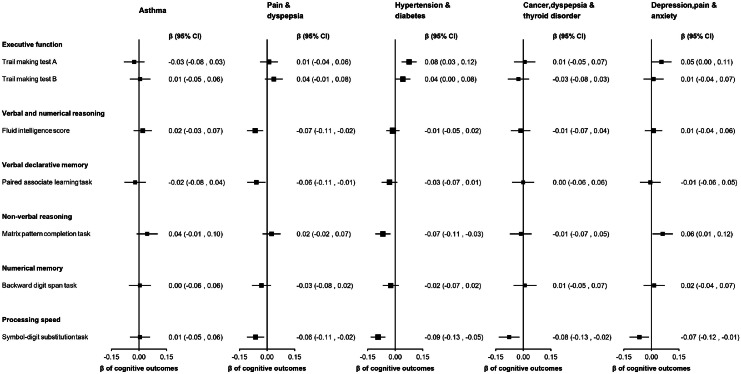
Multivariable linear regression analyses examining associations between disease clusters and standardised cognitive outcomes.

The 'hypertension & diabetes' cluster was associated with poorer performance on TMT A (β=0.08; 95 % CI: 0.03, 0.12), TMT B (β=0.04; 95 % CI: 0.00, 0.08), non-verbal reasoning (β=−0.07; 95 % CI: −0.11, −0.03) and processing speed (β=−0.09; 95 % CI: −0.13, −0.05). The 'pain & dyspepsia' cluster was associated with poorer performance in verbal and numerical reasoning (β=−0.07; 95 % CI: −0.11, −0.02), and verbal declarative memory (β=−0.06; 95 % CI: −0.11, −0.01). The 'depression, pain & anxiety' cluster had associations with non-verbal reasoning (β=0.06; 95 % CI: 0.01, 0.12) and processing speed (β=−0.07; 95 % CI: −0.12, −0.01) (Fig. 4). No other associations between clusters and cognitive outcomes were observed. Although the effect estimates were attenuated, the associations remained consistent after further adjustment for lifestyle factors (**Supplemental Tables 13 and 14**).

Higher scores on the trail making test A or B indicate poorer cognition, while higher scores on the remaining tests indicate better cognition. Trail making tests A and B were log-transformed. All cognitive outcomes were standardised (mean = 0, standard deviation = 1), to facilitate comparison of effect sizes across outcomes. The regression models were adjusted for age, sex, age-squared, age*sex, assessment centre, education, Townsend deprivation index and ethnicity. Point estimates are plotted as squares, with the size of each square proportional to the inverse of the variance of the estimate. Horizontal lines represent 95 % CIs. Each disease cluster was characterised by the top 3 health conditions with the highest probabilities greater than 5 % of contributing to that cluster, excluding conditions for which observed prevalence was equal to or less than that of the total population’s expected prevalence. β= regression co-efficient. CI= confidence interval.

## 4 Discussion

In this large, population-based cross-sectional study of middle- to older-aged adults, we found that multimorbidity was associated with poorer brain health, as indicated by various neuroimaging and cognitive outcomes. A strong dose-response relationship was observed, with increasing multimorbid conditions linked to progressively worse brain health. A disease cluster driven by cardiometabolic conditions displayed the strongest associations with poorer brain health, whilst clusters driven by mental health, respiratory and other conditions displayed inconsistent associations.

Only a limited number of studies have investigated the associations between multimorbidity and neuroimaging outcomes, and all of these have been cross-sectional [10,11,27]. Our findings are consistent with previous research exploring the associations between multimorbidity and neuroimaging outcomes [10,11,27]. Two studies found that multimorbidity was associated with reduced total hippocampal volume [10,11]. In contrast to these studies, we separately analysed the left and right hippocampus and found a stronger association between multimorbidity and left hippocampal volume. This supports evidence suggesting that memory function is more closely linked to the left hippocampus [40]. To our knowledge, only one study has previously explored the effect of different disease clusters on neuroimaging outcomes [27]. This study, involving 36,647 UK-based adults aged 44–81 years, also from UKB, reported associations between clusters characterised by cardiometabolic disorders (CMD), CMD with multiple disorders, and metabolic disorders, with lower total brain and grey matter volumes, as well as higher WMH load [27]. However, direct comparisons with our findings are limited due to methodological differences, including the definition of multimorbidity, the choice of reference group (participants without multimorbidity in our study vs. a “relatively healthy pattern” in the prior study), and the lack of adjustments for key neuroimaging-related confounders such as age-sex interactions, quadratic age terms (age²), scanner position, and head size [27].

We found that multimorbidity was associated with various cognitive domains, including executive functions, verbal declarative memory, and processing speed. A recent systematic review of 16 studies involving 90,606 participants found an association between multimorbidity and an increasing risk of global cognitive decline but reported significant heterogeneity across studies and a lack of a standardised definition for both multimorbidity and cognitive decline [12]. In a previous cross-sectional study of 7,399 UK-based adults, multimorbidity was found to be associated with subjective concentration and memory complaints [41].

We found mixed evidence for effect modification by sex, age and genetic predisposition to dementia. Consistent with previous research, we also found a significant interaction by sex in the association of multimorbidity with global brain volumes, with males showing stronger negative associations than females [27]. However, regional-specific associations with left and total hippocampal volume were stronger in females, whilst the strength of associations with white matter hyperintensities were similar in both females and males. Similar to a previous research, we found no evidence of an interaction by age in the associations of multimorbidity with both neuroimaging and cognitive outcomes [13]. We found limited evidence that genetic predisposition to dementia modified the associations between multimorbidity and brain health. This is consistent with two prospective studies that found no evidence of effect modification by APOE or PRS on multimorbidity and dementia risk [5,32]. However, it contrasts with previous research in the UKB, which found that the presence of multimorbidity and disease clusters were more strongly associated with dementia risk in non-APOE-ε4 carriers [4].

A recent prospective study involving more than 200,000 participants aged ≥60 years in UKB reported that various disease clusters consisting of cardio-metabolic, respiratory, and cancerous conditions were associated with dementia risk [4]. In contrast, only the cardiometabolic cluster was consistently associated with poorer brain health in our study. Hypertension, diabetes, and coronary heart disease are widely recognized as risk factors for dementia and cognitive decline [38,42,43]. Type 2 diabetes has been linked to several features of poorer brain health, such as oxidative stress, the accumulation of advanced glycation end-products, and interference with the degradation of amyloid beta [44]. The negative impact of hypertension and cardiovascular conditions on brain health might be driven by reductions in cerebral blood flow and oxygen supply, increased inflammation, or disruption of the blood-brain barrier [45].

We identified a disease cluster primarily driven by mental health conditions, including depression and anxiety. Depression has been identified as a leading modifiable risk factor for dementia in the 2020 Lancet Commission on dementia prevention [42]. However, we found no association between the mental health cluster and neuroimaging outcomes. Previous studies have found that depression is related to poorer cognitive function and cognitive decline [46]. We found the cluster was associated with poorer performance on two of the seven cognitive outcomes. It is possible that the associations between depression and poorer cognitive function could be due to reverse causation, whereby prodromal dementia increases the likelihood of developing depressive symptoms [47].

Meta-analyses have found that respiratory conditions and reduced pulmonary function are associated with dementia risk [48]. In our study, a cluster driven by respiratory conditions was associated with increased white matter volume and white matter hyperintensities, but was not associated with other neuroimaging or cognitive outcomes. Previous research suggested that impaired pulmonary function could lead to systemic inflammation, oxidative stress and cerebral vessel damage, which might lead to increased cerebrovascular pathology [49]. The other two disease clusters were largely characterised by a miscellaneous set of conditions and included ‘pain and dyspepsia’ and ‘cancer, dyspepsia and thyroid disorders’ as the main drivers. Both generally displayed no associations with neuroimaging outcomes, and were inconsistently associated with cognitive outcomes.

Multimorbidity is highly heterogeneous, with different combinations of conditions likely to influence brain outcomes in distinct ways [3,14]. The multimorbidity clusters identified in our study offer insights into how specific disease combinations are differently associated with neuroimaging and cognitive outcomes. These findings highlight the need for tailored disease management strategies to protect brain health in individuals with multimorbidity. While hypothesis-generating, further research is needed to validate these clusters in other cohorts and evaluate their predictive value for cognitive and brain outcomes across diverse populations.

Our findings demonstrate a particularly strong and consistent association between the cardiometabolic disease cluster and poorer brain health, which is consistent with previous research in this field [32,42,44,45]. Notably, this cluster was identified using a data-driven approach in our study, rather than relying on a pre-defined cardiometabolic group, thus providing robust evidence of associations through a hypothesis-free methodology. This emphasises that the relationship between multimorbidity and poorer brain health is not simply due to the presence of multiple conditions or a random pattern of diseases. Instead, it suggests that cardiometabolic conditions play a central role in driving the associations between multimorbidity and poorer brain health.

Our study extends previous research on multimorbidity and brain health by adopting novel methodological approaches and integrating recent advancements in the field. First, to our knowledge, this is the first study to evaluate the relationship between multimorbidity and brain health by incorporating both neuroimaging measures and validated domain-specific cognitive outcomes within the same study population [28]. Investigating both brain structure and cognition provides a more comprehensive understanding of dementia risk, as both are key aspects of neurodegenerative processes. Second, it is the largest study to date investigating these associations, leveraging an extensive range of phenotypic and genetic data. The large sample size enhances statistical power and reduces the risk of spurious findings, particularly in disease cluster analyses. Third, unlike earlier studies, which analysed multimorbidity presence as a single entity, we examined disease clusters to identify specific combinations of conditions most strongly associated with brain health outcomes [10,11,13]. Fourth, while previous UK Biobank studies stratified multimorbidity analyses by APOE ε4 status, we extended this by including PRS as an additional measure of genetic susceptibility to dementia [27]. Since non-APOE genetic factors are also important contributors to dementia risk, investigating potential effect modification by PRS provides further insights into whether multimorbidity impacts brain health differently across genetic risk profiles [31,32]. Finally, we implemented a more rigorous confounder adjustment strategy, accounting for key neuroimaging-related variables and including interaction terms for age and sex in our multivariable models, as recommended by recent UK Biobank neuroimaging studies, ensuring a more reliable assessment of the relationship between multimorbidity and brain health [33,35].

This study also has several limitations. The chronic conditions used to define multimorbidity were self-reported, making them prone to recall bias and potentially leading to an underestimation of multimorbidity prevalence. However, these self-reported conditions were further verified by a trained nurse during a verbal interview, with uncertain responses matched to corresponding health conditions using a coding tree [50]. Additionally, the low representation of non-white participants limits the generalisability of our findings to other ethnic groups. Furthermore, participants in the UK Biobank are, on average, healthier than the general population, and the imaging sample is a subset of the full cohort [51]. To ensure participant safety and successful completion of the full MRI protocol, additional eligibility criteria were applied for participation in the imaging sub-study [16]. Consequently, participants in the imaging sub-sample are, on average, healthier compared to the full cohort [52]. As a result, the prevalence of certain health conditions might be lower than expected, which could attenuate the strength of associations observed. A future direction would be to investigate whether these findings replicate in other populations. The study's cross-sectional design also precludes conclusions about causality, and longitudinal research is needed to determine how multimorbidity influences brain health over time.

In the present study, multimorbidity was associated with poorer brain health, as evidenced by various neuroimaging and cognitive markers. Particularly strong associations were observed in individuals with cardio-metabolic disease clusters. Given that the UKB participants are relatively healthy compared to the general population, we still found more than 1/3rd of the participants had multimorbidity [51]. Current recommendations for dementia risk reduction typically focus on long-term conditions individually, while we found that the co-existence of these conditions is highly prevalent and strongly associated with brain health [42]. These findings offer additional insights into the potential link between multimorbidity and the future risk of dementia. Further research into the influence of multimorbidity, especially disease clusters, on brain health over time is warranted.

## Consent statement

UK Biobank received ethical approval from the National Health Service (NHS) North West Centre for Research Ethics Committee (Ref: 11/NW/0382). All participants provided electronically signed consent for their data to be used in health-related research.

## Data sharing statement

The UK Biobank Resource holds the data used in this article. Approved researchers can request access to this data through an application to the UK Biobank resource (www.ukbiobank.ac.uk/register-apply).

## Declaration of generative AI and AI-assisted technologies

The authors declare that no generative AI or AI-assisted technologies were used in the writing, or preparation of this manuscript.

## Funding

Elżbieta Kuźma was supported by the Nicolaus and Margrit Langbehn Foundation. Other authors declared no funding or support for the submitted work.

## CRediT authorship contribution statement

**Shehab Uddin Al Abid:** Writing – review & editing, Writing – original draft, Visualization, Formal analysis. **Catherine M Calvin:** Writing – review & editing, Validation, Methodology. **Danial Qureshi:** Writing – review & editing, Visualization, Validation. **Michele Veldsman:** Writing – review & editing, Supervision, Conceptualization. **Elżbieta Kuźma:** Writing – review & editing, Supervision, Conceptualization. **Thomas J. Littlejohns:** Writing – review & editing, Supervision, Resources, Methodology, Data curation, Conceptualization.

## Declaration of competing interest

The authors declare the following financial interests/personal relationships which may be considered as potential competing interests:

Elzbieta Kuzma reports a relationship with Nicolaus and Margrit Langbehn Foundation that includes: funding grants. If there are other authors, they declare that they have no known competing financial interests or personal relationships that could have appeared to influence the work reported in this paper.

## Appendix Supplementary materials

Image, application 1
