# Supplementary material for: Association of multimorbidity and disease clusters with neuroimaging and cognitive outcomes in UK Biobank
Source: J Prev Alzheimers Dis. 2025 May 26;12(7):100208. doi: 10.1016/j.tjpad.2025.100208 (PMC12321637; doi:10.1016/j.tjpad.2025.100208)
Supplement: Supplementary file 1 [file mmc1.docx]

**Association of multimorbidity and disease clusters with neuroimaging and cognitive outcomes in UK Biobank.**

**Supplemental Material**

**Contents**

[**eFigure 1. Study flow chart of identifying eligible participants for analysis.** 3](#_Toc193143227)

[**eTable 1. List of UK Biobank codes for pre-existing neurological conditions.** 4](#_Toc193143228)

[**eTable 2. List of 39 conditions used to define multimorbidity and their prevalence in the analytic sample, overall and by the presence of multimorbidity** 5](#_Toc193143229)

[**eTable 3. List of UK Biobank codes for brain MRI measures and cognitive test measures.** 7](#_Toc193143230)

[**Supplementary Methods: UK Biobank cognitive tests** 8](#_Toc193143231)

[**eTable 4. List of UK Biobank codes for covariates.** 10](#_Toc193143232)

[**eFigure 2. Multivariable linear regression analyses examining association between multimorbidity and A) standardised neuroimaging outcomes and B) standardised cognitive outcomes.** 11](#_Toc193143233)

[**eTable 5. Multivariable linear regression analyses examining the association between multimorbidity and (A) standardised neuroimaging outcomes and (B) standardised cognitive outcomes, with additional adjustment for lifestyle factors** 13](#_Toc193143234)

[**eTable 6. Multivariable linear regression analyses examining the association between the number of multimorbidities, standardised neuroimaging measures, and standardised cognitive test scores.** 14](#_Toc193143235)

[**eTable 7. Multivariable linear regression analyses examining the association between the number of multimorbidities, standardised neuroimaging measures, and standardised cognitive test scores, with additional adjustment for lifestyle factors** 16](#_Toc193143236)

[**eFigure 3. Interaction effect of genetic predispositions for dementia on the association between multimorbidity and standardised neuroimaging outcomes.** 18](#_Toc193143237)

[**eFigure 4. Interaction effect of age and sex on the association between multimorbidity and standardised neuroimaging outcomes.** 20](#_Toc193143238)

[**eFigure 5. Interaction effect of genetic predispositions for dementia on the association between multimorbidity and standardised cognitive outcomes.** 22](#_Toc193143239)

[**eFigure 6. Interaction effect of age and sex on the association between multimorbidity and standardised cognitive outcomes.** 24](#_Toc193143240)

[**eTable 8. Model selection statistics for latent class solution for individuals with multimorbidity in the training sample** 26](#_Toc193143241)

[**eFigure 7. Elbow plot of the model selection statistics for latent class solution for individuals with multimorbidity in the training sample.** 27](#_Toc193143242)

[**eTable 9. Five-class cluster solution of disease using latent class analysis for individuals with multimorbidity in the training sample.** 28](#_Toc193143243)

[**eTable 10. Probabilities and Observed vs Expected Ratios for 38 conditions within 5 clusters.** 29](#_Toc193143244)

[**eTable 11. Multivariable linear regression analyses examining association between disease clusters and standardised neuroimaging outcomes in the test sample.** 31](#_Toc193143245)

[**eTable 12. Multivariable linear regression analyses examining association between disease clusters and standardised cognitive outcomes in the test sample.** 32](#_Toc193143246)

[**eTable 13. Multivariable linear regression analyses examining association between disease clusters and standardised neuroimaging outcomes, with additional adjustment for lifestyle factors** 33](#_Toc193143247)

[**eTable 14. Multivariable linear regression analyses examining association between disease clusters and standardised cognitive outcomes, with additional adjustment for lifestyle factors** 34](#_Toc193143248)

## **eFigure 1. Study flow chart of identifying eligible participants for analysis.**

Participants with available neuroimaging data in UK Biobank: (n=45,001) (extracted on *15/Jun/2023*).

*Participants were excluded due to:*

1. Pre-existing neurological conditions: participants had following conditions during baseline assessments (n=1,008):
 *Stroke (n=392); Dementia/ Alzheimer’s (n=14); Traumatic head injury (n=73), Nervous system infection/ Encephalitis/ meningitis (n=149); Cerebral aneurysm/ subdural hematoma (n=17); Parkinson’s disease (n=78); Multiple sclerosis (n=116); Epilepsy (n=* *180), Brain cancer (n=1); Chronic/ degenerative neurological problem (n=5).*2. Outliers of different Brain MRI markers detected by median absolute deviation method: (n=238)

Included study samples meeting inclusion criteria (n= 43,755) )

**Brain MRI data:**

Included study sample (n= 43,160)

Missing value on covariates: (n=595). (Ethnicity, Townsend deprivation index, education)

**Cognitive test data:**

- Trail Making Test A, n= 27,534
- Trail Making Test B, n= 26,891
- Fluid Intelligence, n= 35,971
- Paired Associate Learning, n= 27,842
- Matrix Pattern Completion, n= 27,549
- Backward Digit Span, n= 28,066
- Symbol-digit Substitution, n= 27,563

## **eTable 1. List of UK Biobank codes for pre-existing neurological conditions.**

| **Neurological conditions** | **Code** | **UK Biobank Field ID** | **Description** |
| --- | --- | --- | --- |
| Stroke | 1081 | 20002 | Tree-structured list used by clinic nurses to code non-cancer illnesses.  <http://biobank.ctsu.ox.ac.uk/crystal/coding.cgi?id=6> |
| Subarachnoid haemorrhage | 1086 |  |  |
| Brain haemorrhage | 1491 |  |  |
| Ischaemic stroke | 1583 |  |  |
| Multiple sclerosis | 1261 |  |  |
| Parkinsons disease | 1262 |  |  |
| Epilepsy | 1264 |  |  |
| Dementia/Alzheimer’s/ Cognitive impairment | 1263 |  |  |
| Neurological injury/ Trauma | 1240 |  |  |
| Head injury | 1266 |  |  |
| Brain abscess/ Intracranial abscess | 1245 |  |  |
| Encephalitis | 1246 |  |  |
| Meningitis | 1247 |  |  |
| Chronic/ Degenerative neurological problem | 1258 |  |  |
| Other demyelinating disease (not multiple sclerosis) | 1397 |  |  |
| Subdural haemorrhage/ Haematoma | 1083 |  |  |
| Cerebral aneurysm | 1425 |  |  |
| Brain cancer / Primary malignant brain tumour | 1032 | 20001 | Tree-structured list used by clinic nurses to code cancers.  <https://biobank.ndph.ox.ac.uk/showcase/coding.cgi?id=3> |

## **eTable 2. List of 39 conditions used to define multimorbidity and their prevalence in the analytic sample, overall and by the presence of multimorbidity**

| **Serial no.** | **Long-term condition** | **Without multimorbidity (%)^a^** | **With multimorbidity (%)^b^** | **Total (% of population)^c^** |
| --- | --- | --- | --- | --- |
|  |  | **N=** **28,821** | **N=14,339** | **N=43,160** |
| 1 | Hypertension | 2,634 (9.1%) | 6,091 (42.5%) | 8,725 (20.2%) |
| 2 | Painful condition | 1,892 (6.6%) | 6,105 (42.6%) | 7,997 (18.5%) |
| 3 | Cancer (except brain cancer) | 1,811 (6.3%) | 2,846 (19.8%) | 4,657 (10.8%) |
| 4 | Treated dyspepsia | 738 (2.6%) | 3,517 (24.5%) | 4,255 (9.9%) |
| 5 | Asthma | 1,047 (3.6%) | 2,772 (19.3%) | 3,819 (8.8%) |
| 6 | Depression | 516 (1.8%) | 2,306 (16.1%) | 2,822 (6.5%) |
| 7 | Thyroid disorders | 646 (2.2%) | 1,794 (12.5%) | 2,440 (5.7%) |
| 8 | Psoriasis or eczema | 396 (1.4%) | 1,833 (12.8%) | 2,229 (5.2%) |
| 9 | Diabetes | 325 (1.1%) | 1,497 (10.4%) | 1,822 (4.2%) |
| 10 | Anxiety & other neurotic, stress related & somatoform disorders | 232 (0.8%) | 1,414 (9.9%) | 1,646 (3.8%) |
| 11 | Migraine | 255 (0.9%) | 1,210 (8.4%) | 1,465 (3.4%) |
| 12 | Prostate disorders | 346 (1.2%) | 1,129 (7.9%) | 1,475 (3.4%) |
| 13 | Coronary heart disease | 206 (0.7%) | 1,021 (7.1%) | 1,227 (2.8%) |
| 14 | Irritable bowel syndrome | 188 (0.7%) | 987 (6.9%) | 1,175 (2.7%) |
| 15 | Rheumatoid arthritis, other inflammatory polyarthropathies & systematic connective tissue disorders | 198 (0.7%) | 740 (5.2%) | 938 (2.2%) |
| 16 | Osteoporosis | 195 (0.7%) | 557 (3.9%) | 752 (1.7%) |
| 17 | Glaucoma | 179 (0.6%) | 522 (3.6%) | 701 (1.6%) |
| 18 | Diverticular disease of intestine | 82 (0.3%) | 589 (4.1%) | 671 (1.6%) |
| 19 | Atrial fibrillation | 139 (0.5%) | 485 (3.4%) | 624 (1.4%) |
| 20 | Chronic obstructive pulmonary disease | 56 (0.2%) | 382 (2.7%) | 438 (1.0%) |
| 21 | Chronic sinusitis | 49 (0.2%) | 339 (2.4%) | 388 (0.9%) |
| 22 | Endometriosis | 42 (0.1%) | 214 (1.5%) | 256 (0.6%) |
| 23 | Inflammatory bowel disease | 67 (0.2%) | 158 (1.1%) | 225 (0.5%) |
| 24 | Transient Ischaemic Attack | 35 (0.1%) | 182 (1.3%) | 217 (0.5%) |
| 25 | Treated constipation | 31 (0.1%) | 159 (1.1%) | 190 (0.4%) |
| 26 | Chronic fatigue syndrome | 21 (0.1%) | 131 (0.9%) | 152 (0.4%) |
| 27 | Bronchiectasis | 22 (0.1%) | 140 (1.0%) | 162 (0.4%) |
| 28 | Chronic kidney disease | 13 (0.0%) | 81 (0.6%) | 94 (0.2%) |
| 29 | Meniere’s disease | 23 (0.1%) | 70 (0.5%) | 93 (0.2%) |
| 30 | Viral hepatitis | 27 (0.1%) | 67 (0.5%) | 94 (0.2%) |
| 31 | Heart failure | 12 (0.0%) | 71 (0.5%) | 83 (0.2%) |
| 32 | Schizophrenia (and related non-organic psychosis) or Bipolar disorder | 16 (0.1%) | 63 (0.4%) | 79 (0.2%) |
| 33 | Pernicious anemia | 10 (0.0%) | 59 (0.4%) | 69 (0.2%) |
| 34 | Polycystic ovary | 8 (0.0%) | 48 (0.3%) | 56 (0.1%) |
| 35 | Chronic liver disease | 11 (0.0%) | 44 (0.3%) | 55 (0.1%) |
| 36 | Peripheral vascular disease | 3 (0.0%) | 19 (0.1%) | 22 (0.1%) |
| 37 | Alcohol problems | 1 (0.0%) | 23 (0.2%) | 24 (0.1%) |
| 38 | Anorexia or bulimia | 5 (0.0%) | 16 (0.1%) | 21 (0.0%) |
| 39 | Other psychoactive substance misuse | 0 | 0 | 0 |

*Notes:*

^a:^ indicates the prevalence of the particular long-term conditions among those who did not have multimorbidity (n=28,821)
^b:^ indicates the prevalence of the particular long-term conditions among those who had multimorbidity (n=14,339)
^c:^ indicates the prevalence of the particular long-term conditions among all participants (n=43,160)

## **eTable 3. List of UK Biobank codes for brain MRI measures and cognitive test measures.**

| **Categories** | **UK Biobank Field ID** | **Description** |
| --- | --- | --- |
| **Brain MRI measures** | | |
| Grey matter volume | 25006 | volume of grey matter (from T1 brain image) |
| White matter volume | 25008 | volume of white matter (from T1 brain image) |
| Total brain volume | 25010 | volume of brain, grey+white, from T1 brain image |
| WMH volume | 25781 | total volume of white matter hyperintensities (from T1 and T2 FLAIR images) |
| Left hippocampal volume | 25019 | volume of left hippocampus (from T1 brain image) |
| Right hippocampal volume | 25020 | volume of right hippocampus (from T1 brain image) |
| Assessment centre | 54 | UK Biobank assessment centre, at which participant underwent imaging |
| Head size | 25000 | volumetric scaling from the T1 head image to standard space |
| Scanner position | 25759 | location of the head and the radio-frequency receive coil in the scanner of participants while undergoing brain MRI |
| **Cognitive test measures** | | |
| Trail Making Test A | 6348 | duration to complete numeric path (trail #1) |
| Trail Making Test B | 6350 | duration to complete alphanumeric path (trail #2) |
| Fluid Intelligence | 20016 | number of correct answers given to the 13 fluid intelligence questions within alloted 2 minutes. |
| Paired Associate Learning | 20197 | number of word pairs correctly associated out of ten attempts. |
| Matrix Pattern Completion | 6373 | number of puzzles correctly solved in 3 minutes |
| Backward Digit Span | 4282 | longest number correctly recalled during the numeric memory test (maximum 12 digit) |
| Symbol-digit Substitution | 23324 | number of symbols correctly matched to digits by the participant in 60 seconds |

## **Supplementary Methods: UK Biobank cognitive tests**

1. **Trail Making Test:**

The Trail Making Test assessed executive function and consisted of two parts, each beginning with a practice trial. In Part A, participants saw a screen with numbers 1-25 arranged randomly and were instructed to touch the numbers in numerical order. In Part B, numbers 1-13 and letters A-L were arranged randomly, and participants were instructed to alternate between touching numbers in numerical order and letters in alphabetical order (i.e., 1-A-2-B-3-C). Participants were encouraged to work as quickly and accurately as possible. If an error was made, the screen flashed red, and the participant had to correct the mistake before continuing. The score was based on the time, in deciseconds, taken to complete each part.

1. **Fluid Intelligence Test:**
   The Fluid Intelligence Test was designed to assess verbal and numerical reasoning. Participants were required to answer 13 multiple-choice questions within 2 minutes. Each question was displayed at the top of the screen, with three to five possible answer options presented below. Participants were instructed to select the answer they believed to be correct or choose ‘Do not know’ or ‘Prefer not to answer.’ Any participants who did not answer all questions within the 2-minute time limit were assigned a score of zero for each unattempted question.
2. **Paired Associate Learning:**

The Paired Associate Learning test assessed verbal declarative memory. Participants were told they would see word pairs and should try to remember them for a later test. They were shown twelve word pairs for 30 seconds (learning phase). Afterward, participants completed another test. Then, they were shown the first word of each pair (the target word) and asked to select the word it was paired with from four options, or choose “Prefer not to answer.” There were 10 questions in total, and the score was based on the number of correct answers.

1. **Matrix Pattern Completion:**

The Matrix Pattern Completion test assessed non-verbal reasoning. Participants were shown a matrix with a missing piece in the lower-right corner and asked to deduce the missing piece based on the pattern's logical structure. They had to identify the correct missing piece from 6 to 8 alternatives. Participants completed three practice items before starting the test, which included 15 items of increasing difficulty. The score was based on the number of correct answers within 3 minutes.

1. **Backward Digit Span:**
   A backward digit span task assessed numerical memory. Participants watched a video demonstration before viewing a two-digit number briefly on the screen. After a short delay, they were asked to recall the number in reverse order. The sequence length increased with each correct recall. If a participant made an error, they were shown a new sequence of the same length. The task ended when the participant failed to recall two sequences of the same length or correctly recalled a 12-digit number. The score was the maximum number of digits correctly recalled in reverse order.
2. **Symbol-digit Substitution:**

A Symbol-digit Substitution test assessed processing speed. Participants were shown a key that paired symbols (top row) with numbers (bottom row). Below the key was a row of symbols. Their task was to enter the corresponding number using a keypad. After a practice trial, participants were instructed to work quickly and accurately. The score was the number of correct symbol-digit matches made in 60 seconds.

## **eTable 4. List of UK Biobank codes for covariates.**

| **Covariates** | **UK Biobank Field ID** | **Description** |
| --- | --- | --- |
| Age (years) | 21003 | Derived from date of birth and date of attending assessment centre  <https://biobank.ndph.ox.ac.uk/ukb/field.cgi?id=21003> |
| Sex | 31 | Acquired from central registry, updated by participant.  <https://biobank.ndph.ox.ac.uk/ukb/field.cgi?id=31> |
| Education | 6138 | Touchscreen questionnaire “Which of the following qualifications do you have? (You can select more than one)”.  <https://biobank.ndph.ox.ac.uk/ukb/field.cgi?id=6138> |
| Ethnicity | 21000 | An amalgam of sequential branching questions asked during the initial assessment centre visit as part of the touchscreen questionnaire.  <https://biobank.ndph.ox.ac.uk/ukb/field.cgi?id=21000> |
| Townsend deprivation index | 22189 | The Townsend Deprivation Index is computed just before a participant joins the UK Biobank, utilizing data from the preceding national census output areas. Each participant receives a score corresponding to the output area where their postcode is situated.  <https://biobank.ndph.ox.ac.uk/ukb/field.cgi?id=22189> |
| Smoking status | 20116 | Participants self-reported their current/past smoking status in the touchscreen questionnaire.  <https://biobank.ndph.ox.ac.uk/ukb/field.cgi?id=20116> |
| Alcohol intake | 20117 | Participants self-reported their current/past alcohol drinking status in the touchscreen questionnaire. <https://biobank.ndph.ox.ac.uk/ukb/field.cgi?id=20117> |
| Body mass index (kg/m^2^) | 21001 | Body mass index (BMI; kg/m^2^) was derived from weight (kg) using scales and standing height (metres) measured during the physical examination.  <https://biobank.ndph.ox.ac.uk/ukb/field.cgi?id=21001> |

## **eFigure 2. Multivariable linear regression analyses examining association between multimorbidity and A) standardised neuroimaging outcomes and B) standardised cognitive outcomes.**


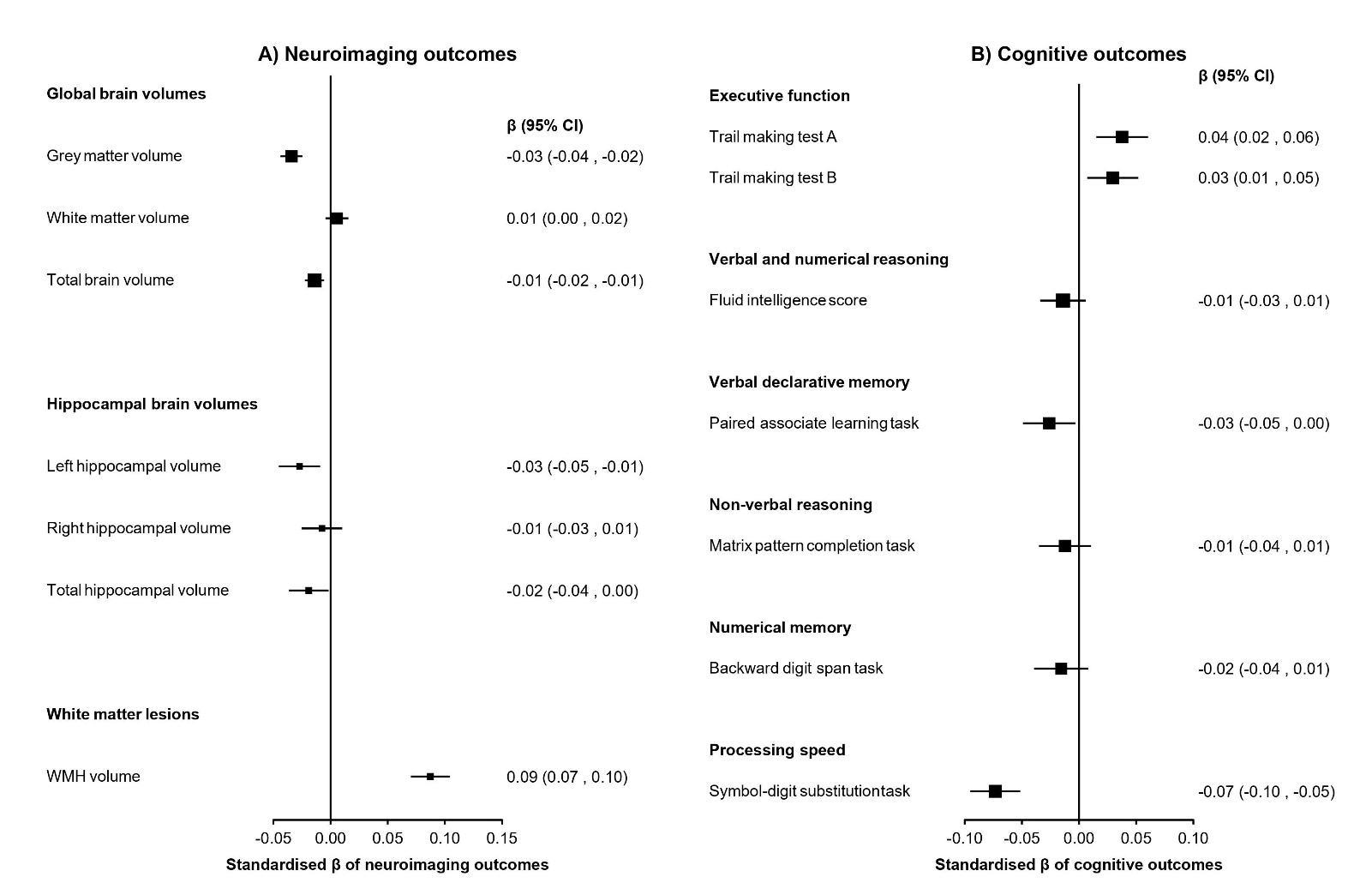


*Notes:* For neuroimaging measures, lower WMH volume indicates better brain health, whereas lower volumes are indicative of poorer brain health for all other neuroimaging outcomes. As for cognitive test scores, higher scores on the trail making test A or B indicate poorer cognition, while higher scores on the remaining tests indicate better cognition.

WMH, Trail making test A and B were log-transformed. All brain and cognitive outcomes were standardised (mean = 0, standard deviation = 1), to facilitate comparison of effect sizes across outcomes. Standardised regression coefficients (β) are reported from individual regression models for all outcomes. The regression models were adjusted for age, sex, age-squared, age*sex, assessment centre, education, Townsend deprivation index, and ethnicity. The neuroimaging outcomes model were additionally adjusted for scanner position and head size. Point estimates are plotted as squares, with the size of each square proportional to the inverse of the variance of the estimate. Horizontal lines represent 95% CIs.

β=regression co-efficient. CI=confidence interval. WMH=White matter hyperintensity.

## **eTable 5. Multivariable linear regression analyses examining the association between multimorbidity and (A) standardised neuroimaging outcomes and (B) standardised cognitive outcomes, with additional adjustment for lifestyle factors**

**A. Neuroimaging outcomes**

|  | **No Multimorbidity** | **Multimorbidity** |
| --- | --- | --- |
|  | **n= 28,821** | **n= 14,339** |
| **Global brain volumes** |  |  |
| Grey matter volume | Ref | **-0.02 (-0.03, -0.01)** |
| White matter volume | Ref | 0.00 (-0.01, 0.01) |
| Total brain volume | Ref | -0.01 (-0.02, 0.00) |
| **Hippocampal brain volumes** |  |  |
| Left hippocampal volume | Ref | **-0.02 (-0.04, 0.00)** |
| Right hippocampal volume | Ref | 0.00 (-0.02, 0.02) |
| Total hippocampal volume | Ref | -0.01 (-0.03, 0.01) |
| **White matter lesions** |  |  |
| WMH volume | Ref | **0.06 (0.04, 0.07)** |

**B. Cognitive outcomes**

|  | **No Multimorbidity** | **Multimorbidity** |
| --- | --- | --- |
|  | **n= 28,821** | **n= 14,339** |
| **Executive function** |  |  |
| Trail making test A | Ref | **0.04 (0.02, 0.06)** |
| Trail making test B | Ref | **0.02 (0.00, 0.05)** |
| **Verbal & numerical reasoning** |  |  |
| Fluid intelligence score | Ref | -0.01 (-0.03, 0.01) |
| **Verbal declarative memory** |  |  |
| Paired associate learning task | Ref | -0.01 (-0.03, 0.02) |
| **Non-verbal reasoning** |  |  |
| Matrix pattern completion task | Ref | 0.00 (-0.03, 0.02) |
| **Numerical memory** |  |  |
| Backward digit span task | Ref | 0.00 (-0.02, 0.03) |
| **Processing speed** |  |  |
| Symbol-digit substitution task | Ref | **-0.06 (-0.08, -0.04)** |

*Notes:* For neuroimaging measures, lower WMH volume indicates better brain health, whereas lower volumes are indicative of poorer brain health for all other neuroimaging outcomes. As for cognitive test scores, higher scores on the trail making test A or B indicate poorer cognition, while higher scores on the remaining tests indicate better cognition.

WMH, Trail making tests A and B were log-transformed. All brain and cognitive outcomes were standardised (mean = 0, standard deviation = 1), to facilitate comparison of effect sizes across outcomes. Standardised regression coefficients (β) are reported from individual regression models for all outcomes. Regression coefficients with a p-value < 0.05 are marked in bold. The regression models were adjusted for age, sex, age-squared, age*sex, assessment centre, education, Townsend deprivation index, ethnicity, smoking status, alcohol intake and BMI. The neuroimaging outcomes model was additionally adjusted for scanner position and head size. β=regression co-efficient. CI=confidence interval. WMH=White matter hyperintensity.

## **eTable 6. Multivariable linear regression analyses examining the association between the number of multimorbidities, standardised neuroimaging measures, and standardised cognitive test scores.**

**A. Neuroimaging outcomes**

|  | **0-1 condition** | **2 conditions** | **3 conditions** | **≥4 conditions** | ***P* for** |
| --- | --- | --- | --- | --- | --- |
|  | **n= 28,821** | **n= 7,744** | **n= 3,887** | **n= 2,708** | **trend test** |
| **Global brain volumes** |  |  |  |  |  |
| Grey matter volume | 0.01 (0.01, 0.02) | -0.01 (-0.02, -0.00) | -0.02 (-0.04, -0.01) | -0.06 (-0.08, -0.04) | <0.001 |
| White matter volume | 0.00 (-0.01, 0.00) | 0.00 (-0.01, 0.02) | 0.01 (-0.01, 0.02) | 0.00 (-0.02, 0.02) | 0.62 |
| Total brain volume | 0.00 (-0.00, 0.01) | 0.00 (-0.01, 0.01) | -0.01 (-0.02, 0.01) | -0.03 (-0.05, -0.02) | <0.001 |
| **Hippocampal brain volumes** |  |  |  |  |  |
| Left hippocampal volume | 0.01 (-0.00, 0.02) | 0.00 (-0.02, 0.02) | -0.04 (-0.06, -0.01) | -0.05 (-0.09, -0.02) | <0.001 |
| Right hippocampal volume | 0.00 (-0.01, 0.01) | 0.00 (-0.02, 0.02) | 0.00 (-0.03, 0.02) | -0.03 (-0.06, 0.02) | 0.30 |
| Total hippocampal volume | 0.01 (-0.00, 0.02) | 0.00 (-0.02, 0.02) | -0.02 (-0.05, 0.00) | -0.05 (-0.08, -0.01) | <0.01 |
| **White matter lesions** |  |  |  |  |  |
| WMH volume | -0.03 (-0.04, -0.02) | 0.03 (0.01, 0.04) | 0.08 (0.05, 0.11) | 0.12 (0.09, 0.15) | <0.001 |

**B. Cognitive outcomes**

|  | **0-1 condition** | **2 conditions** | **3 conditions** | **≥4 conditions** | ***P* for** |  |
| --- | --- | --- | --- | --- | --- | --- |
|  | **n= 28,821** | **n= 7,744** | **n= 3,887** | **n= 2,708** | **trend test** |  |
| **Executive function** |  |  |  |  |  | |
| Trail making test A | -0.01 (-0.02, 0.00) | 0.02 (-0.01, 0.04) | 0.03 (-0.01, 0.06) | 0.05 (0.01, 0.10) | <0.01 | |
| Trail making test B | -0.01 (-0.02, 0.00) | 0.01 (-0.02, 0.03) | 0.02 (-0.02, 0.05) | 0.06 (0.02, 0.11) | <0.01 | |
| **Verbal & numerical reasoning** |  |  |  |  |  | |
| Fluid intelligence score | 0.00 (-0.01, 0.02) | 0.01 (-0.01, 0.03) | -0.02 (-0.05, 0.02) | -0.06 (-0.10, -0.03) | <0.01 | |
| **Verbal declarative memory** |  |  |  |  |  | |
| Paired associate learning task | 0.01 (-0.00, 0.02) | 0.00 (-0.03, 0.03) | -0.02 (-0.06, 0.01) | -0.07 (-0.11, -0.02) | <0.01 | |
| **Non-verbal reasoning** |  |  |  |  |  | |
| Matrix pattern completion task | 0.00 (-0.01, 0.02) | 0.00 (-0.03, 0.02) | -0.01 (-0.05, -0.02) | -0.02 (-0.06, 0.03) | 0.70 | |
| **Numerical memory** |  |  |  |  |  | |
| Backward digit span task | 0.01 (-0.01, 0.02) | 0.00 (-0.03, 0.02) | 0.00 (-0.04, 0.04) | -0.05 (-0.09, -0.00) | 0.21 | |
| **Processing speed** |  |  |  |  |  | |
| Symbol-digit substitution task | 0.02 (0.01, 0.04) | -0.03 (-0.06, -0.01) | -0.04 (0.07, -0.00) | -0.13 (-0.17, -0.09) | <0.001 | |

*Notes:* All neuroimaging and cognitive outcomes were standardised (mean = 0, standard deviation = 1), to facilitate comparison of effect sizes across outcomes. Standardised mean of all outcomes along with 95% CI are reported. A test for trend across categories of multimorbidity (2, 3, and ≥4 conditions) was conducted following running each regression models. The regression models were adjusted for age, sex, age-squared, age*sex, assessment centre, scanner position, head size, education, Townsend deprivation index and ethnicity. Head size and scanner position were additionally adjusted for in the neuroimaging analyses.

Lower WMH volume indicates better brain health, whereas lower volumes are indicative of poorer brain health for all other neuroimaging outcomes. Higher scores on the trail making test A or B indicate poorer cognition, while higher scores on the remaining tests indicate better cognition. CI=confidence interval. WMH=White matter hyperintensity.

## **eTable 7. Multivariable linear regression analyses examining the association between the number of multimorbidities, standardised neuroimaging measures, and standardised cognitive test scores, with additional adjustment for lifestyle factors**

**A. Neuroimaging outcomes**

|  | **0-1 condition** | **2 conditions** | **3 conditions** | **≥4 conditions** | ***P* for** |
| --- | --- | --- | --- | --- | --- |
|  | **n= 28,821** | **n= 7,744** | **n= 3,887** | **n= 2,708** | **trend test** |
| **Global brain volumes** |  |  |  |  |  |
| Grey matter volume | 0.01 (0.00, 0.01) | 0.00 (-0.01, 0.01) | -0.01 (-0.03, 0.00) | -0.04 (-0.06, -0.02) | <0.001 |
| White matter volume | 0.00 (-0.01, 0.00) | 0.00 (-0.01, 0.01) | 0.01 (-0.01, 0.02) | -0.01 (-0.02, 0.01) | 0.70 |
| Total brain volume | 0.00 (0.00, 0.01) | 0.00 (-0.01, 0.01) | 0.00 (-0.02, 0.01) | -0.02 (-0.04, -0.01) | <0.05 |
| **Hippocampal brain volumes** |  |  |  |  |  |
| Left hippocampal volume | 0.01 (0.00, 0.02) | 0.01 (-0.01, 0.03) | -0.03 (-0.06, 0.00) | -0.04 (-0.08, -0.01) | <0.01 |
| Right hippocampal volume | 0.00 (-0.01, 0.01) | 0.01 (-0.01, 0.03) | 0.00 (-0.03, 0.03) | -0.02 (-0.05, 0.01) | 0.64 |
| Total hippocampal volume | 0.00 (-0.01, 0.01) | 0.01 (-0.01, 0.03) | -0.02 (-0.04, 0.01) | -0.03 (-0.07, 0.00) | 0.08 |
| **White matter lesions** |  |  |  |  |  |
| WMH volume | -0.02 (-0.03, -0.01) | 0.01 (-0.01, 0.03) | 0.06 (0.03, 0.08) | 0.08 (0.05, 0.11) | <0.001 |

**B. Cognitive outcomes**

|  | **0-1 condition** | **2 conditions** | **3 conditions** | **≥4 conditions** | ***P* for** |  |
| --- | --- | --- | --- | --- | --- | --- |
|  | **n= 28,821** | **n= 7,744** | **n= 3,887** | **n= 2,708** | **trend test** |  |
| **Executive function** |  |  |  |  |  | |
| Trail making test A | -0.01 (-0.03, 0.00) | 0.02 (-0.01, 0.04) | 0.02 (-0.01, 0.06) | 0.06 (0.01, 0.10) | <0.01 | |
| Trail making test B | -0.01 (-0.02, 0.00) | 0.01 (-0.02, 0.03) | 0.01 (-0.03, 0.04) | 0.05 (0.00, 0.09) | 0.08 | |
| **Verbal & numerical reasoning** |  |  |  |  |  | |
| Fluid intelligence score | 0.00 (-0.01, 0.02) | 0.02 (-0.01, 0.04) | -0.01 (-0.04, 0.02) | -0.05 (-0.08, -0.01) | <0.05 | |
| **Verbal declarative memory** |  |  |  |  |  | |
| Paired associate learning task | 0.00 (-0.01, 0.02) | 0.01 (-0.02, 0.03) | -0.01 (-0.04, 0.03) | -0.04 (-0.08, 0.01) | 0.31 | |
| **Non-verbal reasoning** |  |  |  |  |  | |
| Matrix pattern completion task | 0.00 (-0.01, 0.02) | 0.00 (-0.03, 0.02) | 0.00 (-0.04, 0.03) | 0.00 (-0.04, 0.04) | 0.98 | |
| **Numerical memory** |  |  |  |  |  | |
| Backward digit span task | 0.00 (-0.01, 0.02) | 0.01 (-0.02, 0.03) | 0.01 (-0.02, 0.05) | -0.02 (-0.07, 0.02) | 0.71 | |
| **Processing speed** |  |  |  |  |  | |
| Symbol-digit substitution task | 0.02 (0.01, 0.03) | -0.02 (-0.05, 0.00) | -0.02 (-0.06, 0.01) | -0.11 (-0.15, -0.07) | <0.001 | |

*Notes:* All neuroimaging and cognitive outcomes were standardised (mean = 0, standard deviation = 1), to facilitate comparison of effect sizes across outcomes. Standardised mean of all outcomes along with 95% CI are reported. A test for trend across categories of multimorbidity (2, 3, and ≥4 conditions) was conducted following running each regression models. The regression models were adjusted for age, sex, age-squared, age*sex, assessment centre, scanner position, head size, education, Townsend deprivation index, ethnicity, smoking status, alcohol intake and BMI. Head size and scanner position were additionally adjusted for in the neuroimaging analyses.

Lower WMH volume indicates better brain health, whereas lower volumes are indicative of poorer brain health for all other neuroimaging outcomes. Higher scores on the trail making test A or B indicate poorer cognition, while higher scores on the remaining tests indicate better cognition. CI=confidence interval. WMH=White matter hyperintensity.

## **eFigure 3. Interaction effect of genetic predispositions for dementia on the association between multimorbidity and standardised neuroimaging outcomes.**


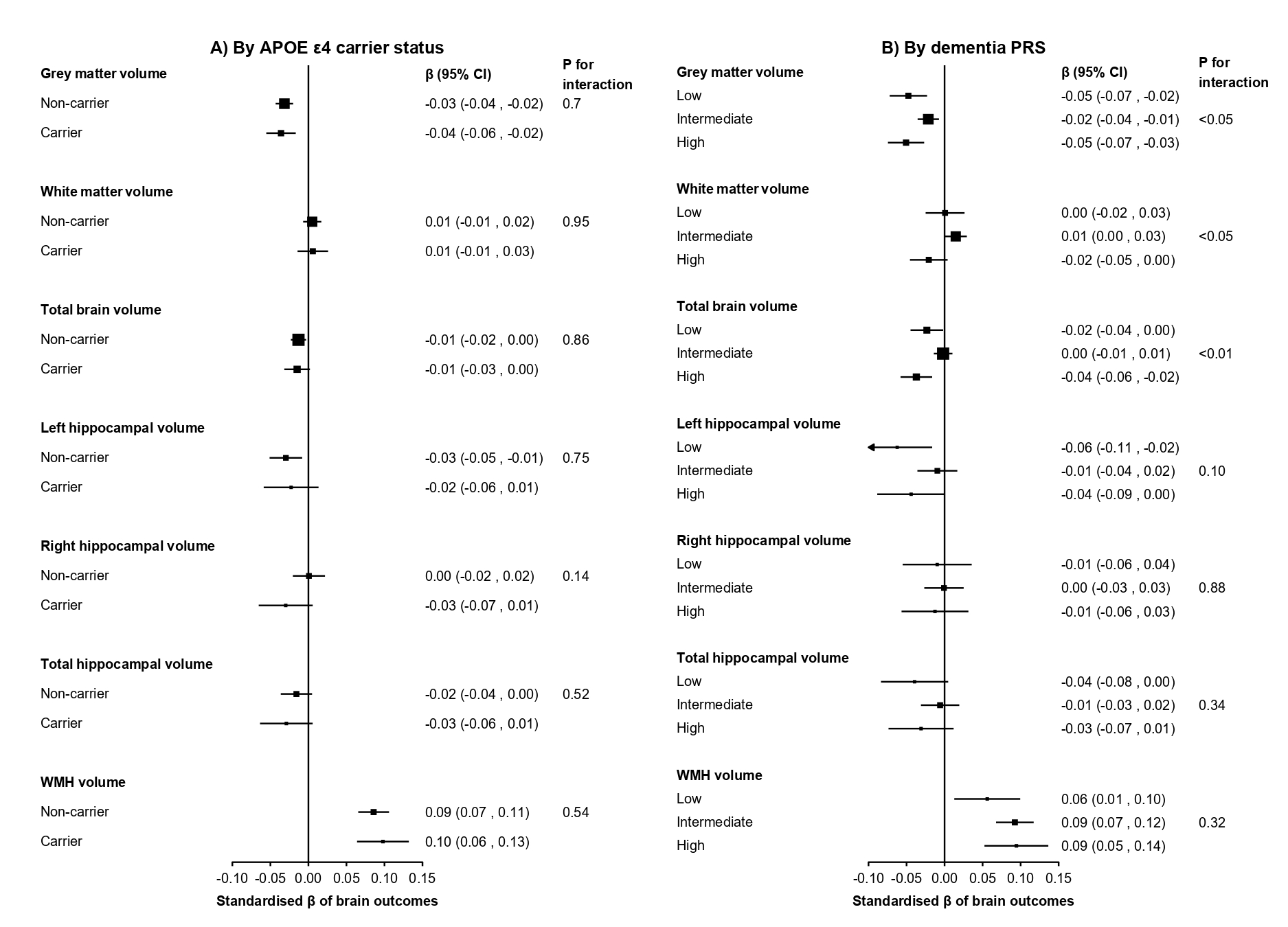


*Notes:* Lower WMH volume indicates better brain health, whereas lower volumes are indicative of poorer brain health for all other brain outcomes. WMH was log-transformed. All brain outcomes were standardised (mean = 0, standard deviation = 1), to facilitate comparison of effect sizes across outcomes.

Interaction terms were added into the fully adjusted model, adjusted for age, sex, age-squared, age*sex, scanner position, head size, assessment centre, education, Townsend deprivation index, and ethnicity. P-values for interaction were obtained from the likelihood ratio tests after adding the interaction term.

Point estimates are plotted as squares, with the size of each square proportional to the inverse of the variance of the estimate. Horizontal lines represent 95% CIs. Since the polygenic risk score was generated for Europeans, the analysis was restricted to only European/White ethnic groups to assess interactions with non-APOE polygenic risk scores (PRS).

APOE= Apolipoprotein. β=regression co-efficient. CI=confidence interval. WMH=white matter hyperintensity. PRS=polygenic risk score

## **eFigure 4. Interaction effect of age and sex on the association between multimorbidity and standardised neuroimaging outcomes.**


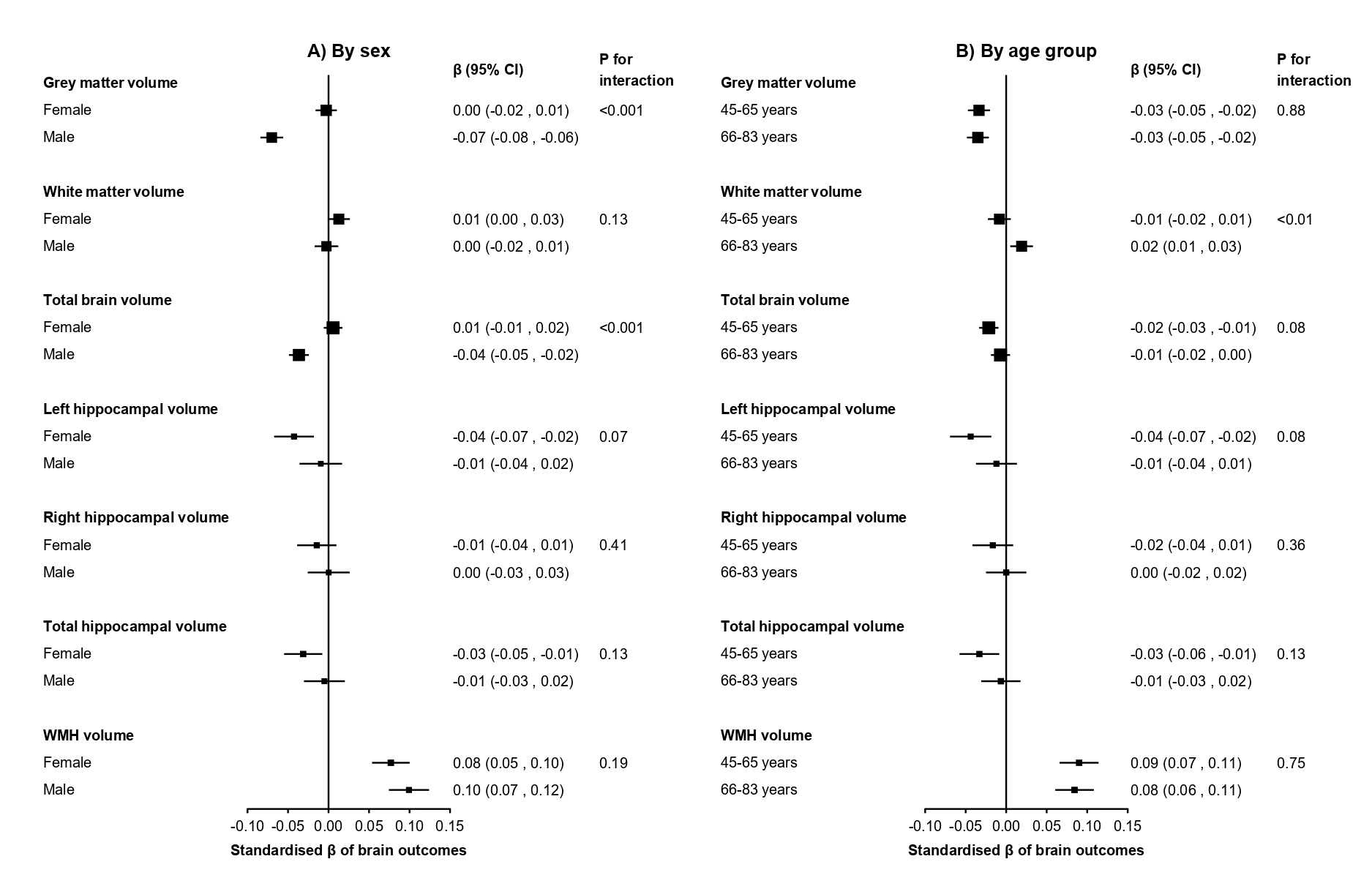


*Notes:* Lower WMH volume indicates better brain health, whereas lower volumes are indicative of poorer brain health for all other brain outcomes. WMH was log-transformed. All brain outcomes were standardised (mean = 0, standard deviation = 1), to facilitate comparison of effect sizes across outcomes.

Interaction terms were added into the fully adjusted model, adjusted for age, sex, age-squared, age*sex, scanner position, head size, assessment centre, education, Townsend deprivation index, and ethnicity. P-values for interaction were obtained from the likelihood ratio tests after adding the interaction term.

Point estimates are plotted as squares, with the size of each square proportional to the inverse of the variance of the estimate. Horizontal lines represent 95% CIs.

β=regression co-efficient. CI=confidence interval. WMH=white matter hyperintensity.

## **eFigure 5. Interaction effect of genetic predispositions for dementia on the association between multimorbidity and standardised cognitive outcomes.**


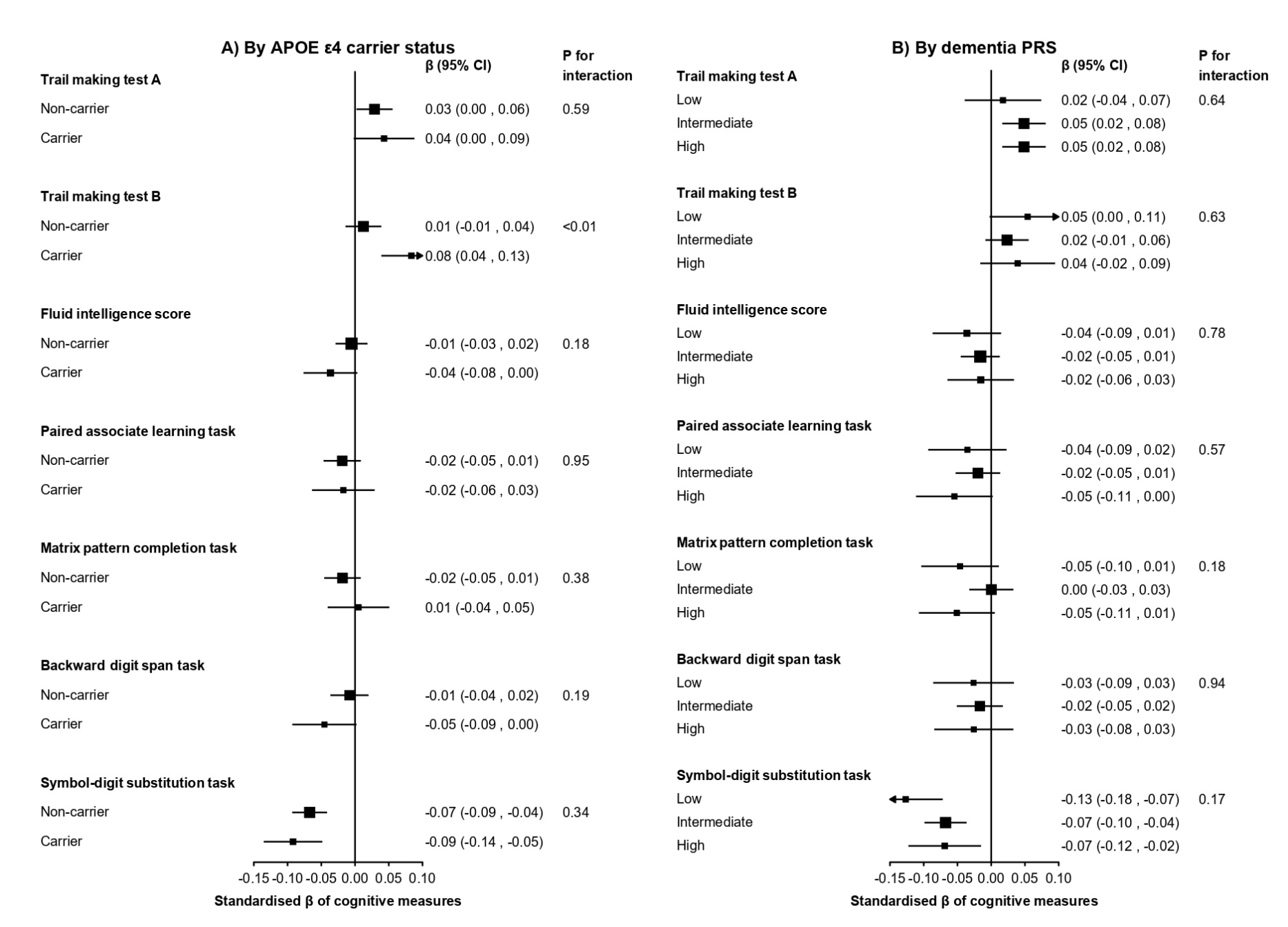


*Notes:* Higher scores on the trail making test A or B indicate poorer cognition, while higher scores on the remaining tests indicate better cognition. Trail making test A and B were log-transformed. All cognitive outcomes were standardised (mean = 0, standard deviation = 1), to facilitate comparison of effect sizes across outcomes.

Interaction terms were added into the fully adjusted model, adjusted for age, sex, age-squared, age*sex, assessment centre, education, Townsend deprivation index, and ethnicity. P-values for interaction were obtained from the likelihood ratio tests after adding the interaction term.

Point estimates are plotted as squares, with the size of each square proportional to the inverse of the variance of the estimate. Horizontal lines represent 95% CIs. Since the polygenic risk score was generated for Europeans, the analysis was restricted to only European/White ethnic groups to assess interactions with non-APOE polygenic risk scores (PRS).

APOE= Apolipoprotein. β=regression co-efficient. CI=confidence interval. PRS=polygenic risk score

## **eFigure 6. Interaction effect of age and sex on the association between multimorbidity and standardised cognitive outcomes.**


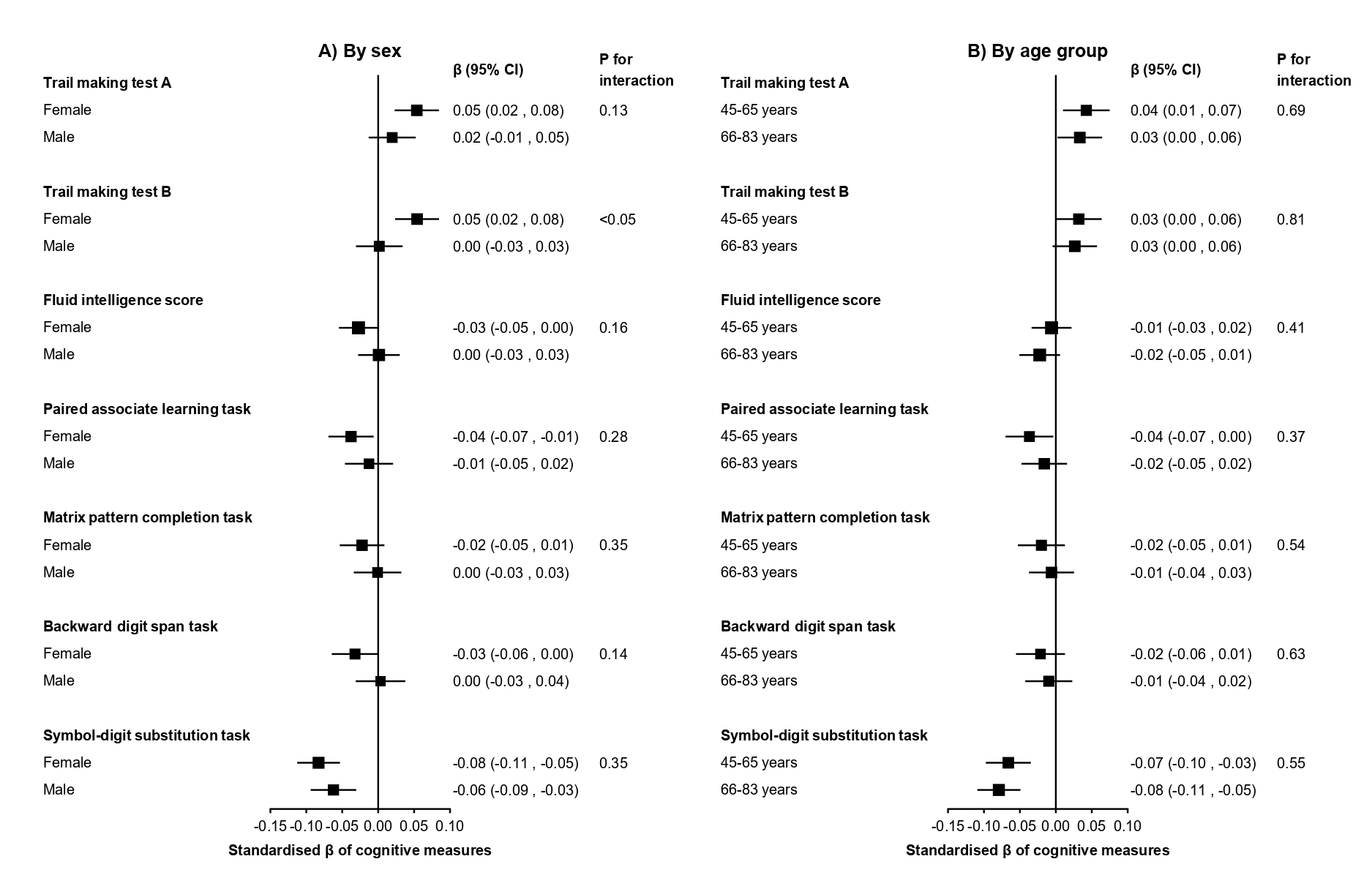


*Notes:* Higher scores on the trail making test A or B indicate poorer cognition, while higher scores on the remaining tests indicate better cognition. Trail making test A and B were log-transformed. All cognitive outcomes were standardised (mean = 0, standard deviation = 1), to facilitate comparison of effect sizes across outcomes.

Interaction terms were added into the fully adjusted model, adjusted for age, sex, age-squared, age*sex, assessment centre, education, Townsend deprivation index, and ethnicity. P-values for interaction were obtained from the likelihood ratio tests after adding the interaction term.

Point estimates are plotted as squares, with the size of each square proportional to the inverse of the variance of the estimate. Horizontal lines represent 95% CIs.

β=regression co-efficient. CI=confidence interval.

## **eTable 8. Model selection statistics for latent class solution for individuals with multimorbidity in the training sample**

| **Model** | **Log likelihood** | **df** | **BIC** | **ABIC** | **CAIC** | **Likelihood ratio** | **Entropy** |
| --- | --- | --- | --- | --- | --- | --- | --- |
| Model 1 | -75671 | 10000 | 151693 | 151572 | 151731 | 21428 | - |
| Model 2 | -74740 | 9961 | 150190 | 149946 | 150267 | 19567 | 0.434 |
| Model 3 | -74428 | 9922 | 149925 | 149557 | 150041 | 18942 | 0.646 |
| Model 4 | -74172 | 9883 | 149773 | 149280 | 149928 | 18430 | 0.656 |
| Model 5 | -73942 | 9844 | 149671 | 149054 | 149865 | 17969 | 0.719 |
| Model 6 | -73830 | 9805 | 149806 | 149066 | 150039 | 17745 | 0.68 |
| Model 7 | -73689 | 9766 | 149885 | 149021 | 150157 | 17465 | 0.776 |
| Model 8 | -73551 | 9727 | 149968 | 148980 | 150279 | 17188 | 0.746 |
| Model 9 | -73446 | 9688 | 150117 | 149004 | 150467 | 16977 | 0.766 |
| Model 10 | -73255 | 9649 | 150095 | 148859 | 150484 | 16596 | 0.934 |
| Model 11 | -73323 | 9610 | 150590 | 149230 | 151018 | 16733 | NaN |
| Model 12 | -73146 | 9571 | 150594 | 149110 | 151061 | 16377 | 0.804 |

*Notes:* ABIC= adjusted Bayesian information criterion. BIC= Bayesian information criterion. CAIC= consistent Akaike's information criterion. df=degree of freedom.

## **eFigure 7. Elbow plot of the model selection statistics for latent class solution for individuals with multimorbidity in the training sample.**


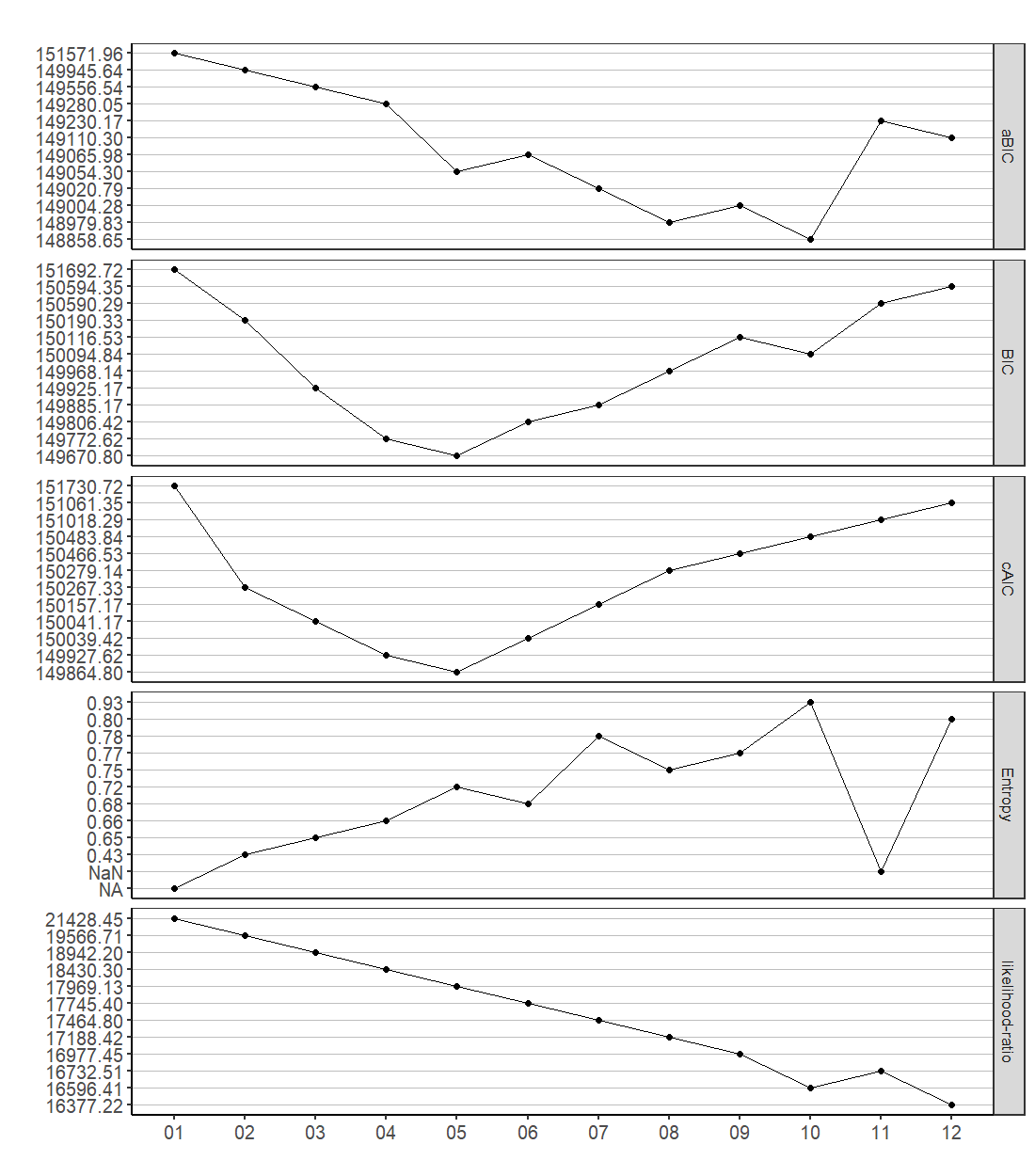


*Notes:* df=degree of freedom. BIC= Bayesian information criterion. ABIC= adjusted Bayesian information criterion. CAIC= consistent Akaike's information criterion.

## **eTable 9. Five-class cluster solution of disease using latent class analysis for individuals with multimorbidity in the training sample.**

| **Cluster** | **% of training sample (n = 10,338)** | **Male (%)** | **Lead condition** | **Subsidiary condition 1** | **Subsidiary condition 2** |
| --- | --- | --- | --- | --- | --- |
| 1 | 15.36 | 45.33 | Asthma (100%) | - | - |
| 2 | 20.99 | 44.85 | Pain (100%) | Dyspepsia (27.9%) | - |
| 3 | 32.15 | 57.82 | Hypertension (100%) | Diabetes (20.6%) | - |
| 4 | 14.04 | 45.64 | Cancer (40.2%) | Dyspepsia (30.9%) | Thyroid disorder (25.0%) |
| 5 | 17.46 | 30.69 | Depression (47.5%) | Pain (47.4%) | Anxiety (33.4%) |

*Notes:* Each disease cluster was characterized by the top 3 health conditions with the highest probabilities greater than 5% of contributing to that cluster, excluding conditions for which observed prevalence was equal to or less than that of the total population’s expected prevalence. The percentages indicate the prevalence of the condition within the cluster, ie, for asthma, 100% of individuals within the cluster had asthma.

Blank cells had the conditions for which their observed prevalence was equal to or less than that of the expected prevalence of the total population.

## **eTable 10. Probabilities and Observed vs Expected Ratios for 38 conditions within 5 clusters.**

| **Condition** |  | **Asthma** | | **Pain & dyspepsia** | | **Hypertension & diabetes** | | **Cancer, dyspepsia & thyroid disorders** | | **Depression, pain & anxiety** | |
| --- | --- | --- | --- | --- | --- | --- | --- | --- | --- | --- | --- |
|  | **Expected** | **P** | **O/E** | **P** | **O/E** | **P** | **O/E** | **P** | **O/E** | **P** | **O/E** |
| Alcohol problems | 0.002 | 0.00 | 0.35 | 0.00 | 0.00 | 0.00 | 0.75 | 0.00 | 0.25 | 0.00 | 2.30 |
| Anorexia or bulimia | 0.001 | 0.00 | 1.00 | 0.00 | 0.10 | 0.00 | 0.20 | 0.00 | 0.00 | 0.01 | 5.40 |
| Anxiety | 0.097 | 0.02 | 0.25 | 0.02 | 0.16 | 0.04 | 0.40 | 0.04 | 0.42 | 0.33 | 3.45 |
| Asthma | 0.195 | 1.00 | 5.13 | 0.00 | 0.00 | 0.09 | 0.48 | 0.00 | 0.00 | 0.15 | 0.79 |
| Atrial Fibrillation | 0.033 | 0.02 | 0.62 | 0.04 | 1.16 | 0.04 | 1.10 | 0.07 | 2.20 | 0.00 | 0.15 |
| Bronchiectasis | 0.01 | 0.02 | 2.14 | 0.01 | 0.72 | 0.01 | 0.58 | 0.02 | 1.97 | 0.01 | 0.59 |
| Cancer | 0.198 | 0.15 | 0.75 | 0.19 | 0.96 | 0.20 | 1.00 | 0.40 | 2.03 | 0.10 | 0.49 |
| Chronic fatigue syndrome | 0.009 | 0.01 | 1.02 | 0.00 | 0.20 | 0.00 | 0.18 | 0.01 | 1.27 | 0.03 | 2.81 |
| Chronic Kidney disease | 0.006 | 0.00 | 0.75 | 0.00 | 0.27 | 0.01 | 1.57 | 0.01 | 1.05 | 0.00 | 0.78 |
| Chronic liver disease | 0.003 | 0.00 | 0.00 | 0.00 | 0.63 | 0.00 | 0.80 | 0.01 | 2.37 | 0.00 | 0.80 |
| COPD | 0.026 | 0.05 | 1.73 | 0.02 | 0.74 | 0.02 | 0.90 | 0.04 | 1.44 | 0.02 | 0.61 |
| Coronary Heart Disease | 0.07 | 0.04 | 0.56 | 0.05 | 0.70 | 0.11 | 1.61 | 0.11 | 1.56 | 0.02 | 0.31 |
| Depression | 0.16 | 0.08 | 0.50 | 0.04 | 0.25 | 0.08 | 0.50 | 0.09 | 0.59 | 0.47 | 2.97 |
| Diabetes | 0.106 | 0.06 | 0.58 | 0.05 | 0.51 | 0.21 | 1.94 | 0.12 | 1.12 | 0.04 | 0.35 |
| Diverticular disease | 0.042 | 0.03 | 0.69 | 0.05 | 1.25 | 0.03 | 0.76 | 0.05 | 1.15 | 0.05 | 1.23 |
| Endometriosis | 0.016 | 0.01 | 0.61 | 0.02 | 1.31 | 0.00 | 0.31 | 0.01 | 0.50 | 0.03 | 2.16 |
| Glaucoma | 0.036 | 0.02 | 0.56 | 0.03 | 0.91 | 0.04 | 1.23 | 0.07 | 1.98 | 0.01 | 0.34 |
| Heart Failure | 0.004 | 0.00 | 0.85 | 0.00 | 0.68 | 0.01 | 1.70 | 0.01 | 2.30 | 0.00 | 0.00 |
| Hypertension | 0.426 | 0.23 | 0.54 | 0.26 | 0.60 | 1.00 | 2.35 | 0.03 | 0.07 | 0.19 | 0.45 |
| Inflammatory bowel disease | 0.011 | 0.01 | 1.09 | 0.01 | 1.07 | 0.01 | 0.72 | 0.02 | 1.87 | 0.01 | 0.57 |
| Irritable bowel syndrome | 0.07 | 0.04 | 0.63 | 0.06 | 0.92 | 0.02 | 0.23 | 0.06 | 0.86 | 0.17 | 2.42 |
| Meniere’s disease | 0.005 | 0.00 | 0.16 | 0.01 | 1.10 | 0.00 | 0.76 | 0.01 | 1.74 | 0.01 | 1.04 |
| Migraine | 0.087 | 0.04 | 0.50 | 0.12 | 1.41 | 0.03 | 0.35 | 0.07 | 0.77 | 0.17 | 2.00 |
| Osteoporosis | 0.039 | 0.03 | 0.78 | 0.03 | 0.70 | 0.02 | 0.60 | 0.10 | 2.56 | 0.03 | 0.86 |
| Painful condition | 0.425 | 0.32 | 0.75 | 1.00 | 2.35 | 0.23 | 0.54 | 0.06 | 0.13 | 0.47 | 1.11 |
| Peripheral vascular disease | 0.001 | 0.00 | 2.20 | 0.00 | 0.00 | 0.00 | 2.40 | 0.00 | 2.00 | 0.00 | 0.00 |
| Pernicious anaemia | 0.004 | 0.01 | 1.78 | 0.00 | 0.90 | 0.00 | 0.25 | 0.01 | 1.80 | 0.01 | 1.53 |
| Polycystic ovarian syndrome | 0.003 | 0.00 | 0.50 | 0.01 | 1.70 | 0.00 | 0.17 | 0.00 | 0.00 | 0.01 | 3.20 |
| Prostate disorders | 0.079 | 0.06 | 0.72 | 0.10 | 1.25 | 0.08 | 1.07 | 0.13 | 1.67 | 0.03 | 0.36 |
| Psoriasis or eczema | 0.124 | 0.16 | 1.29 | 0.14 | 1.16 | 0.06 | 0.48 | 0.11 | 0.88 | 0.18 | 1.46 |
| Rheumatoid arthritis | 0.052 | 0.04 | 0.79 | 0.04 | 0.84 | 0.04 | 0.80 | 0.12 | 2.29 | 0.03 | 0.63 |
| Schizophrenia | 0.005 | 0.00 | 0.62 | 0.00 | 0.34 | 0.00 | 0.72 | 0.01 | 1.44 | 0.01 | 1.56 |
| Sinusitis | 0.024 | 0.01 | 0.33 | 0.03 | 1.28 | 0.00 | 0.19 | 0.01 | 0.35 | 0.06 | 2.68 |
| Thyroid disorder | 0.123 | 0.10 | 0.83 | 0.10 | 0.78 | 0.10 | 0.81 | 0.25 | 2.03 | 0.11 | 0.88 |
| Transient Ischaemic Attack | 0.012 | 0.00 | 0.36 | 0.01 | 0.86 | 0.02 | 1.32 | 0.02 | 1.46 | 0.01 | 0.73 |
| Treated constipation | 0.011 | 0.01 | 0.51 | 0.02 | 1.45 | 0.00 | 0.42 | 0.01 | 0.91 | 0.02 | 1.92 |
| Treated dyspepsia | 0.246 | 0.20 | 0.83 | 0.28 | 1.13 | 0.18 | 0.73 | 0.31 | 1.26 | 0.29 | 1.16 |
| Viral hepatitis | 0.004 | 0.00 | 0.85 | 0.00 | 0.48 | 0.00 | 1.23 | 0.01 | 1.78 | 0.00 | 1.08 |

*Notes:* Abbreviations: O/E= Observed/Expected. P= Probability. COPD= Chronic Obstructive Pulmonary Disease. TIA=Transient Ischemic Attack.

Yellow cells flag O/E > 1; of these the 3 highest probability conditions with >0.10 are marked as blue.

## **eTable 11. Multivariable linear regression analyses examining association between disease clusters and standardised neuroimaging outcomes in the test sample.**

| **Neuroimaging outcomes** | **No multimorbidity** | **Asthma (100%)** | **Pain (63.7%) & Dyspepsia (34.7%)** | **Hypertension (76.2%) & Diabetes (24.2%)** | **Cancer (100%) & Hypertension (43.4%)** | **Depression (100%), Pain (43.7%) & Anxiety (36.0%)** |
| --- | --- | --- | --- | --- | --- | --- |
|  | **n= 28,821** | **n= 625** | **n= 1,307** | **n= 1,202** | **n=626** | **n=541** |
| **Global brain volumes** |  |  |  |  |  |  |
| Grey matter volume | Ref | 0.01 (-0.02, 0.05) | 0.02 (-0.01, 0.04) | **-0.13 (-0.16, -0.10)** | -0.04 (-0.07, 0.00) | -0.03 (-0.07, 0.01) |
| White matter volume | Ref | 0.01 (-0.03, 0.05) | 0.01 (-0.02, 0.04) | 0.00 (-0.03, 0.03) | 0.01 (-0.03, 0.04) | -0.01 (-0.05, 0.03) |
| Total brain volume | Ref | 0.01 (-0.02, 0.05) | 0.01 (-0.01, 0.04) | -0.06 (-0.09, -0.04) | -0.02 (-0.05, 0.02) | -0.02 (-0.06, 0.01) |
| **Hippocampal brain volumes** |  |  |  |  |  |  |
| Left hippocampal volume | Ref | -0.05 (-0.12, 0.02) | -0.04 (-0.09, 0.01) | **-0.06 (-0.11, -0.01)** | -0.03 (-0.10, 0.04) | -0.00 (-0.08, 0.07) |
| Right hippocampal volume | Ref | -0.02 (-0.08, 0.05) | -0.00 (-0.05, 0.05) | -0.03 (-0.08, 0.02) | -0.02 (-0.09, 0.05) | 0.00 (-0.07, 0.08) |
| Total hippocampal volume | Ref | -0.03 (-0.10, 0.03) | -0.02 (-0.07, 0.03) | **-0.05 (-.10, -0.00)** | -0.03 (-0.09, 0.04) | -0.00 (-0.07, 0.07) |
| **White matter lesions** |  |  |  |  |  |  |
| WMH volume | Ref | 0.02 (-0.04, 0.09) | -0.00 (-0.05, 0.04) | **0.24 (0.19, 0.29)** | **0.08 (0.01, 0.14)** | **0.07 (0.00, 0.14)** |

*Notes:* Lower WMH volume indicates better brain health, whereas lower volumes are indicative of poorer brain health for all other brain outcomes. WMH was log-transformed. All brain outcomes were standardised (mean = 0, standard deviation = 1), to facilitate comparison of effect sizes across outcomes. Regression coefficients with a p-value < 0.05 are marked in bold. Standardised beta with 95% CI is reported.

All regression models were adjusted for age, sex, age-squared, age*sex, assessment centre, scanner position, head size, education, Townsend deprivation index and ethnicity.

Each disease cluster was characterized by the top 3 health conditions with the highest probabilities greater than 5% of contributing to that cluster, excluding conditions for which observed prevalence was equal to or less than that of the total population’s expected prevalence. The percentages indicate the prevalence of the condition within the cluster, ie, for asthma, 100% of individuals within the cluster had asthma.

β=regression co-efficient. CI=confidence interval. WMH=White matter hyperintensity.

## **eTable 12. Multivariable linear regression analyses examining association between disease clusters and standardised cognitive outcomes in the test sample.**

| **Cognitive outcomes** | **No multimorbidity** | **Asthma (100%)** | **Pain (63.7%) & Dyspepsia (34.7%)** | **Hypertension (76.2%) & Diabetes (24.2%)** | **Cancer (100%) & Hypertension (43.4%)** | **Depression (100%), Pain (43.7%) & Anxiety (36.0%)** |
| --- | --- | --- | --- | --- | --- | --- |
|  | **n= 28,821** | **n= 625** | **n= 1,307** | **n= 1,202** | **n=626** | **n=541** |
| **Executive function** |  |  |  |  |  |  |
| Trail making test A | Ref | 0.03 (-0.06, 0.12) | 0.06 (-0.01, 0.12) | 0.03 (-0.04, 0.09) | 0.05 (-0.04, 0.13) | **0.12 (0.02, 0.22)** |
| Trail making test B | Ref | 0.02 (-0.07, 0.10) | 0.04 (-0.02, 0.10) | 0.04 (-0.02, 0.11) | 0.06 (-0.03, 0.14) | **0.13 (0.03, 0.22)** |
| **Verbal & numerical reasoning** |  |  |  |  |  |  |
| Fluid intelligence score | Ref | -0.01 (-0.09, 0.06) | -0.04 (-0.09, 0.02) | -0.01 (-0.07, 0.04) | 0.06 (-0.02, 0.14) | -0.06 (-0.14, 0.02) |
| **Verbal declarative memory** |  |  |  |  |  |  |
| Paired associate learning task | Ref | 0.02 (-0.08, 0.11) | -0.03 (-0.09, 0.03) | -0.05 (-0.11, 0.02) | -0.00 (-0.09, 0.08) | -0.02 (-0.12, 0.08) |
| **Non-verbal reasoning** |  |  |  |  |  |  |
| Matrix pattern completion task | Ref | 0.06 (-0.03, 0.15) | -0.04 (-0.10, 0.02) | **-0.08 (-0.15, -0.01)** | -0.02 (-0.11, 0.07) | -0.09 (-0.19, 0.01) |
| **Numerical memory** |  |  |  |  |  |  |
| Backward digit span task | Ref | 0.03 (-0.06, 0.12) | -0.03 (-0.10, 0.03) | -0.07 (-0.14, 0.00) | -0.02 (-0.11, 0.07) | -0.03 (-0.13, 0.07) |
| **Processing speed** |  |  |  |  |  |  |
| Symbol-digit substitution task | Ref | -0.07 (-0.15, 0.02) | **-0.09 (-0.15, 0.03)** | **-0.09 (-0.15, -0.03)** | **-0.09 (-0.18, -0.01)** | **-0.16 (-0.26, -0.07)** |

*Notes:* Higher scores on the trail making test A or B indicate poorer cognition, while higher scores on the remaining tests indicate better cognition. Trail making test A and B were log-transformed. All cognitive outcomes were standardised (mean = 0, standard deviation = 1), to facilitate comparison of effect sizes across outcomes. Regression coefficients with a p-value < 0.05 are marked in bold. Standardised beta with 95% CI are reported. The regression models were adjusted for age, sex, age-squared, age*sex, assessment centre, education, Townsend deprivation index and ethnicity. Each disease cluster was characterized by the top 3 health conditions with the highest probabilities greater than 5% of contributing to that cluster, excluding conditions for which observed prevalence was equal to or less than that of the total population’s expected prevalence. The percentages indicate the prevalence of the condition within the cluster, ie, for asthma, 100% of individuals within the cluster had asthma. β=regression co-efficient. CI=confidence interval.

## **eTable 13. Multivariable linear regression analyses examining association between disease clusters and standardised neuroimaging outcomes, with additional adjustment for lifestyle factors**

| **Neuroimaging outcomes** | **No multimorbidity** | **Asthma (100%)** | **Pain (100%) & Dyspepsia (27.9%)** | **Hypertension (100%) & Diabetes (20.6%)** | **Cancer (40.2%), Dyspepsia (30.9%) & Thyroid disorder (25.0%)** | **Depression (47.5%), Pain (47.4%) & Anxiety (33.4%)** |
| --- | --- | --- | --- | --- | --- | --- |
|  | **n= 28,821** | **n= 1,542** | **n= 2,107** | **n= 3,227** | **n=1,409** | **n=1,753** |
| **Global brain volumes** |  |  |  |  |  |  |
| Grey matter volume | Ref | 0.01 (-0.01, 0.04) | 0.01 (-0.01, 0.04) | **-0.07 (-0.09, -0.05)** | -0.02 (-0.04, 0.01) | 0.02 (-0.01, 0.04) |
| White matter volume | Ref | 0.03 (0.00, 0.05) | -0.01 (-0.03, 0.01) | 0.01 (-0.01, 0.03) | -0.02 (-0.04, 0.01) | 0.00 (-0.02, 0.03) |
| Total brain volume | Ref | 0.02 (0.00, 0.04) | 0.00 (-0.02, 0.02) | **-0.03 (-0.05, -0.02)** | -0.02 (-0.04, 0.00) | 0.01 (-0.01, 0.03) |
| **Hippocampal brain volumes** |  |  |  |  |  |  |
| Left hippocampal volume | Ref | -0.02 (-0.07, 0.02) | -0.02 (-0.05, 0.02) | -0.03 (-0.06, 0.00) | 0.00 (-0.05, 0.05) | 0.02 (-0.02, 0.06) |
| Right hippocampal volume | Ref | 0.01 (-0.03, 0.06) | 0.00 (-0.03, 0.04) | -0.03 (-0.06, 0.00) | 0.01 (-0.04, 0.05) | 0.04 (0.00, 0.08) |
| Total hippocampal volume | Ref | 0.00 (-0.05, 0.04) | -0.01 (-0.04, 0.03) | **-0.03 (-0.07, 0.00)** | 0.00 (-0.04, 0.05) | 0.03 (-0.01, 0.08) |
| **White matter lesions** |  |  |  |  |  |  |
| WMH volume | Ref | 0.02 (-0.02, 0.06) | -0.01 (-0.05, 0.02) | **0.18 (0.15, 0.21)** | -0.01 (-0.06, 0.03) | -0.02 (-0.06, 0.02) |

*Notes:* Lower WMH volume indicates better brain health, whereas lower volumes are indicative of poorer brain health for all other brain outcomes. WMH was log-transformed. All neuroimaging outcomes were standardised (mean = 0, standard deviation = 1), to facilitate comparison of effect sizes across outcomes. Regression coefficients with a p-value < 0.05 are marked in bold. Standardised beta with 95% CI is reported. Regression coefficients with a p-value < 0.05 are marked in bold. All regression models were adjusted for age, sex, age-squared, age*sex, assessment centre, scanner position, head size, education, Townsend deprivation index, ethnicity, smoking status, alcohol intake and BMI.

Each disease cluster was characterized by the top 3 health conditions with the highest probabilities greater than 5% of contributing to that cluster, excluding conditions for which observed prevalence was equal to or less than that of the total population’s expected prevalence. The percentages indicate the prevalence of the condition within the cluster, ie, for asthma, 100% of individuals within the cluster had asthma.

β=regression co-efficient. CI=confidence interval. WMH=White matter hyperintensity.

## **eTable 14. Multivariable linear regression analyses examining association between disease clusters and standardised cognitive outcomes, with additional adjustment for lifestyle factors**

| **Cognitive outcomes** | **No multimorbidity** | **Asthma (100%)** | **Pain (100%) & Dyspepsia (27.9%)** | **Hypertension (100%) & Diabetes (20.6%)** | **Cancer (40.2%), Dyspepsia (30.9%) & Thyroid disorder (25.0%)** | **Depression (47.5%), Pain (47.4%) & Anxiety (33.4%)** |
| --- | --- | --- | --- | --- | --- | --- |
|  | **n= 28,821** | **n= 1,542** | **n= 2,107** | **n= 3,227** | **n=1,409** | **n=1,753** |
| **Executive function** |  |  |  |  |  |  |
| Trail making test A | Ref | -0.03 (-0.08, 0.03) | 0.01 (-0.04, 0.06) | **0.08 (0.03, 0.12)** | 0.00 (-0.05, 0.06) | 0.05 (0.00, 0.11) |
| Trail making test B | Ref | 0.00 (-0.05, 0.06) | 0.03 (-0.01, 0.08) | 0.03 (-0.01, 0.07) | -0.03 (-0.09, 0.03) | 0.01 (-0.05, 0.06) |
| **Verbal & numerical reasoning** |  |  |  |  |  |  |
| Fluid intelligence score | Ref | 0.04 (-0.01, 0.09) | -0.04 (-0.09, 0.00) | 0.01 (-0.03, 0.05) | 0.00 (-0.06, 0.05) | 0.04 (-0.01, 0.09) |
| **Verbal declarative memory** |  |  |  |  |  |  |
| Paired associate learning task | Ref | -0.01 (-0.06, 0.05) | **-0.06 (-0.11, -0.01)** | 0.00 (-0.05, 0.04) | -0.01 (-0.07, 0.05) | 0.00 (-0.06, 0.05) |
| **Non-verbal reasoning** |  |  |  |  |  |  |
| Matrix pattern completion task | Ref | 0.04 (-0.02, 0.09) | 0.01 (-0.03, 0.06) | **-0.07 (-0.11, -0.03)** | -0.02 (-0.08, 0.04) | 0.05 (0.00, 0.11) |
| **Numerical memory** |  |  |  |  |  |  |
| Backward digit span task | Ref | 0.03 (-0.03, 0.09) | -0.02 (-0.08, 0.03) | 0.00 (-0.04, 0.05) | 0.01 (-0.05, 0.07) | 0.02 (-0.03, 0.08) |
| **Processing speed** |  |  |  |  |  |  |
| Symbol-digit substitution task | Ref | 0.01 (-0.04, 0.07) | -0.05 (-0.10, -0.01) | -0.07 (-0.11, -0.03) | -0.07 (-0.13, -0.02) | -0.05 (-0.10, 0.01) |

*Notes:* Higher scores on the trail making test A or B indicate poorer cognition, while higher scores on the remaining tests indicate better cognition. Trail making test A and B were log-transformed. All cognitive outcomes were standardised (mean = 0, standard deviation = 1), to facilitate comparison of effect sizes across outcomes. Regression coefficients with a p-value < 0.05 are marked in bold. Standardised beta with 95% CI is reported.Regression coefficients with a p-value < 0.05 are marked in bold. The regression models were adjusted for age, sex, age-squared, age*sex, assessment centre, education, Townsend deprivation index, ethnicity, smoking status, alcohol intake and BMI.

Each disease cluster was characterized by the top 3 health conditions with the highest probabilities greater than 5% of contributing to that cluster, excluding conditions for which observed prevalence was equal to or less than that of the total population’s expected prevalence. The percentages indicate the prevalence of the condition within the cluster, ie, for asthma, 100% of individuals within the cluster had asthma. β=regression co-efficient. CI=confidence interval.
